# Effects of explicit cueing and ambiguity on the anticipation and experience of a painful thermal stimulus

**DOI:** 10.1371/journal.pone.0183650

**Published:** 2017-08-23

**Authors:** Lincoln M. Tracy, Stephen J. Gibson, Nellie Georgiou-Karistianis, Melita J. Giummarra

**Affiliations:** 1 School of Psychological Sciences and Monash Institute of Cognitive and Clinical Neurosciences, Monash University, Clayton, Victoria, Australia; 2 Caulfield Pain Management & Research Centre, Caulfield Hospital, Caulfield, Victoria, Australia; 3 School of Public Health and Preventive Medicine, Monash University, Melbourne, Victoria, Australia; 4 Institute of Safety, Compensation & Recovery Research, Monash University, Melbourne, Victoria, Australia; Université catholique de Louvain, BELGIUM

## Abstract

Many factors can influence the way in which we perceive painful events and noxious stimuli, but less is known about how pain perception is altered by explicit knowledge about the impending sensation. This study aimed to investigate the impact of explicit cueing on anxiety, arousal, and pain experience during the anticipation and delivery of noxious thermal heat stimulations. Fifty-two healthy volunteers were randomised to receive explicit instructions about visual cue-stimulus temperature pairings, or no explicit instructions about the cue-stimulus pairs. A pain anxiety task was used to investigate the effects of explicit cueing on anticipatory anxiety, pain experience and electrophysiological responses. Participants who received explicit instructions about the cue-stimulus pairs (i.e., the relationship between the colour of the cue and the temperature of the associated stimuli) reported significantly higher subjective anxiety prior to the delivery of the thermal heat stimuli (*p* = .025, partial eta squared = .10). There were no effects of explicit cueing on subsequent pain intensity, unpleasantness, or the electrophysiological response to stimulus delivery. The perceived intensity and unpleasantness of the stimuli decreased across the blocks of the paradigm. In both groups anticipating the ambiguous cue elicited the largest change in electrophysiological arousal, indicating that not knowing the impending stimulus temperature led to increased arousal, compared to being certain of receiving a high temperature thermal stimulus (both *p* < .001). Perceived stimulus intensity varied between ambiguous and non-ambiguous cues, depending on the temperature of the stimulus. Together these findings highlight the impact and importance of explicit cueing and uncertainty in experimental pain studies, and how these factors influence the way healthy individuals perceive and react to noxious and innocuous thermal stimuli.

## Introduction

Pain has been defined as the distressing experience associated with actual or potential tissue damage, involving sensory, emotional, cognitive, and social components [1], and is clearly far more complex and multifaceted than simply the transmission of nociceptive signals. There is a complex, non-linear relationship between nociceptive input and the intensity of pain that is perceived by an individual [2]. Pain experience can almost be conceptualised as a parallel to perceptual illusions, whereby pain perception is influenced by all of the available information, including prior experiences, and what the person expects to happen [3]. This study was designed to examine whether pain experience varies in relation to whether we do, or do not know what to expect about an impending thermal heat stimulus (i.e., the temperature of the stimulus). Understanding how the perception and experience of pain is influenced by certainty and expectations is critically important for experimental pain research.

Due to its complex nature, the quantification and assessment of pain can be challenging. In empirical studies participants are frequently asked to report their pain experience on a rating scale, which is usually linear with semantic anchors of no pain to worst pain at each extreme (e.g., the numerical ratings scale, NRS). This psychophysical method for pain reporting has been used extensively for decades by researchers and clinicians to examine the effects of a range of factors (e.g., pharmacological manipulations) on pain perception [4]. Many researchers also monitor skin conductance and/or heart rate to assess autonomic responses to painful stimuli, with most demonstrating increases in both heart rate [5–9] and skin conductance [10–13] following the delivery of painful stimuli.

Pain can serve as a protective mechanism to warn us of potential or actual danger. However, in some situations, particularly experimental or research situations, avoiding pain is not possible [14]. Under these circumstances the anticipation of pain leads to physiological responses (i.e., increased autonomic arousal, anxiety, and perceived pain intensity of a painful stimulus), which essentially prepare the individual for protective behaviours to promote recuperation [15, 16]. When an individual is presented with the threat of a potentially painful stimulus, there is also typically a surge in sympathetic arousal [17], with increases in blood pressure and the skin conductance response (SCR) compared to a resting state [18–20], which serve to prepare one for action to avoid harm. Inhibitory parasympathetic arousal then elicits a decrease in heart rate (HR), to facilitate orienting responses, focussed attention and sensory processing [21].

Providing specific information about an upcoming event can bring about expectations of potentially painful events i.e., the expectation effect [22–27]. Consequently, a noxious stimulus is perceived as more intense if the stimulus is preceded by a cue that denotes a higher intensity stimulus, compared to if it was preceded by a cue that denotes a lower intensity stimulus [22, 28, 29]. Similar effects can be observed in clinical settings. For example, higher ratings of pain are given during a procedure by patients who were warned (another form of cueing or priming) that they will perceive a “sting” prior to venous blood sampling [30] or the insertion of an intravenous cannula [31], compared to patients who were not warned beforehand.

It is not clear how explicit knowledge of stimulus intensity influences self-reported and electrophysiological arousal during the anticipation and experience of potentially painful stimuli, especially in an experimental study. Understanding the effects of explicit cueing on responses to noxious experimental stimuli is critical as these responses have, and will continue to, affect the interpretation of experimental data. This consideration is important for the design of future experimental pain research studies. For the present study we designed an experimental task that would allow us to investigate the impact of explicit cueing on self-reported anticipatory anxiety, arousal, and thermal (heat) pain experience in response to both ambiguous and unambiguous cues.

Participants were randomised to receive either detailed, explicit instructions about the task (“Hint” group), or very sparse instructions (“No Hint” group), before starting the task. We hypothesised that participants in the “Hint” group, compared to the “No Hint” group, would (a) report increased anticipatory anxiety and display increased electrophysiological arousal (i.e., HR and SCR) during the anticipation of a noxious thermal heat stimulus; (b) provide higher ratings of pain intensity and pain unpleasantness in response the stimuli; and (c) display increased levels of electrophysiological arousal during the experience of thermal stimuli, particularly for thermal stimuli preceded by an ambiguous cue (designed to create an atmosphere of uncertainty and heightened anxiety).

## Method

### Participants

A convenience sample of fifty-two healthy students and young professionals (mean age = 21.9 years, range = 18 to 36 years, 26 females) from Monash University and the surrounding areas participated in the experiment, which was approved by the Monash University Human Research Ethics Committee (project number CF14/1640–2014000772). All participants provided written informed consent. This sample size is similar to samples recruited in prior empirical studies investigating pain and pain anxiety [32, 33]. Potential participants were screened for eligibility against the exclusion criteria, described below, prior to being invited to participate in the study. Data on volunteers who did not meet the inclusion criteria were not recorded. No participants withdrew from the study after providing consent. Potential participants were excluded from the study if they self-reported acute or chronic pain (i.e., pain that is persistent for more than three months); current analgesic or psychotropic medication use; colour blindness; or other medical conditions suspected or known to be associated with pain sensitivity (e.g., diabetes, neurodegenerative diseases, or previous injuries and major trauma). Participants were also excluded if they were identified to be at risk of anxious/depressive disorders and/or suicidal ideation (see ‘Mood Questionnaires’). Prior to the experimental testing session participants were instructed to abstain from alcohol for 24 hours, and caffeinated beverages and nicotine for four hours as these are known to influence pain perception and autonomic arousal. Compliance with these criteria were noted by self-report.

### Electrophysiological data

This study used an 8/35 PowerLab unit and dual BioAmplifier (AD Instruments, Sydney, Australia) for continuous measurement of HR and SCR. For HR, a five-lead electrocardiogram (ECG) was used with disposable, pre-gelled electrodes (35mm diameter, Coviden). The raw ECG signal was filtered with a 0.3 to 20 Hz band-pass filter, sampled at a rate of 2000 Hz, before being smoothed with a Savitzky-Golay filter (window width 155 samples). To measure SCR, finger electrodes (MLT116F) were placed on the ventral surface of the proximal phalanx of the second and fourth fingers of the participants’ non-dominant hand. Prior to the commencement of the experimental task both circuit- and subject-zeroing was performed for SCR to account for inter-individual variability between participants. HR and SCR data were monitored and analysed using LabChart Pro version 7.3.7 software (AD Instruments, Sydney, Australia).

### Thermal stimuli

The Medoc Pathway Pain and Sensory Evaluation System (Medoc Advanced Medical Systems Ltd, Ramat Yishay, Israel) with Medoc Main Station software version 6.3.6.18.1 was utilised to deliver thermal stimulations to participants. The Pathway system allows for exact, controllable delivery of thermal stimuli using the Contact Heat Evoked Potentials (CHEPS) thermode. The CHEPS thermode has a round contact area of 573 mm^2^ (27 mm in diameter) and can produce temperatures between 30°C and 55°C, with the ability to increase in temperature at a rate of 70°C/second. The CHEPS thermode was securely attached to the volar surface of the participant’s dominant arm, 5 cm proximal from the wrist crease with a Velcro strap. The baseline temperature for the thermode throughout the pain anxiety task was 32°C, the same as the “innocuous stimulus”.

### Pain anxiety task

**Fig 1:**
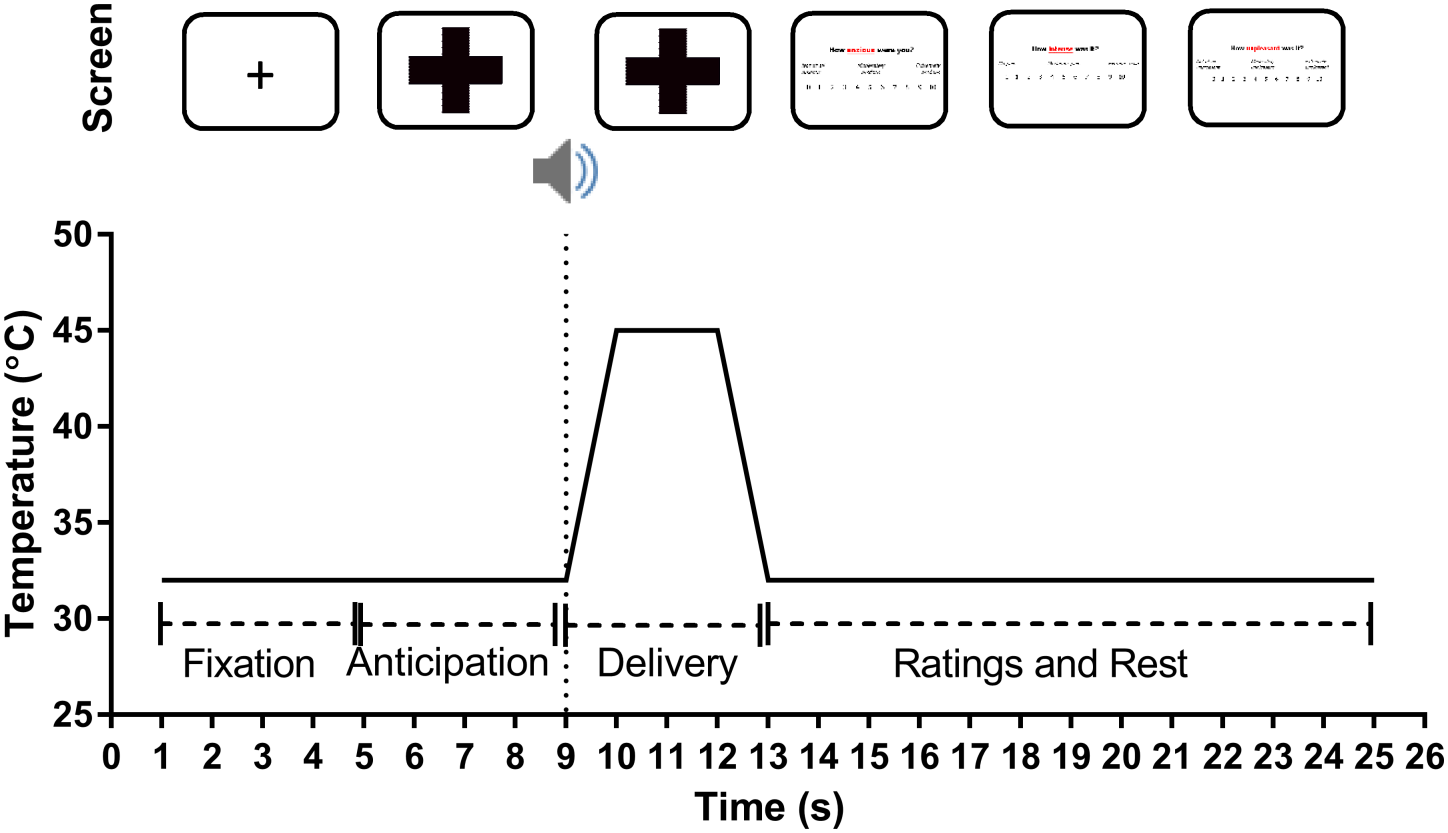
Trial design for the pain anxiety task. Note that although the duration of the stimulus in Fig 1 is listed as four seconds, this includes one second for the thermode to increase from the baseline temperature to the target temperature, and an additional second for the thermode to return to the baseline temperature following the two second application of the stimulus at the target temperature. A 0.5s ‘beep’ signalled the commencement of thermal stimulus delivery. After each stimulus was delivered participants were prompted to provide ratings of anticipatory anxiety, pain intensity, and pain unpleasantness.

The pain anxiety task comprised three blocks, with 24 trials per block. The sequence for an individual trial is provided in Fig 1. All visual cues (i.e., coloured crosses) were presented to participants via a Dell Latitude E6440 laptop computer with a 14” screen, with a standard white background, using SuperLab version 4.5 presentation software. When presented on the laptop computer screen, the coloured crosses were 10.5 cm high and 11.6 cm wide, with a visual angle of 10.91°. Each trial started with a fixation cross (always presented for 4 s), followed by an anticipatory cue (always presented for 4 s), and then the thermal stimulus was always delivered over a four second period. The cues indicated that the temperature of the stimulus would be high (45°C), low-moderate (41°C), innocuous (32°C), or that the stimulus could be any of those three temperatures (i.e., ambiguous). Temperatures were selected based on screening the literature [34], and pilot testing in a separate sample, to identify temperatures that were consistently rated as high (i.e., 6-10/10), low (i.e., 3-5/10), and innocuous (i.e., 0-2/10) in the majority of healthy young adults. The authors were more interested in the effects of explicit cueing and uncertainty on the anticipation and experience of pain, rather than investigating individual differences in sensitivity to pain. Therefore, this study employed a *response*-dependent methodology, where consistent temperatures were used for all participants and the responses to these stimuli were measures, rather than a *stimulus*-dependent methodology [35], where the stimulus temperatures are personalised to individual participant sensitivity. Response-dependent methodologies have previously been used in studies investigating the effect of cueing, fear, and threat on the anticipation and experience of pain [32, 33, 36]. Over the course of each block, each target temperature was delivered eight times in pseudorandomised order. Half of the trials were specific (i.e., they were preceded by an unambiguous cue), while the remaining 50% were preceded by the ambiguous cue. The ambiguous cue was included as a condition where we aimed to create greater anxiety in participants as a consequence of the uncertain nature of the following stimulus. The relationship between the visual cue presented and the temperature of the paired thermal stimulus is summarised in Table 1.

**Table 1:** Relationship between visual cues and stimulus temperatures.

Cue Colour	R	G	B	Stimulus Temperature
Pink	225	0	102	32°C
Orange	247	150	70	41°C
Purple	128	100	162	45°C
Blue	75	172	198	32 or 41 or 45°C

*Note*: Each block comprised 24 stimuli, in which each target temperature was delivered eight times in pseudorandomised order; 50% preceded by their partnered unambiguous cue, while the remaining 50% were preceded by the ambiguous cue. The RGB values refer to the additive colour model in which red, green, and blue are added together to produce a broad array of colours.

Before commencing the experiment, participants were randomly allocated into one of two groups and were given standardised written instructions in a sealed opaque envelope labelled with their individual participant number. Participants were instructed to open the envelope and read the written instructions, ensuring that the experimenter was unable to see what was written on their instructions to ensure the experimenter was blinded to participant allocation. Participants were allowed to read through the instructions as many times as necessary to understand them, but were not allowed to seek clarification about the written instructions from the researcher. When the participant was comfortable with their understanding of the instructions the participant placed the instructions back in the opaque envelope and resealed it, ensuring that the researcher remained blinded to their group allocation for the duration of the session. The envelopes remained resealed until data from all participants had been collected and entered, after which the experimenter was unblinded to group allocations. All participants received the following written instructions: “*In this experiment you will be presented with a series of cues*, *with thermal stimuli following each cue*. *Following each stimulus you will be asked a series of questions relating to how you experienced and perceived the cue and its paired stimulus*”. Participants in the “Hint” group then received a significantly more detailed description of the relationship between the colour of the visual cue and the intensity of the associated thermal stimulus compared to the “No Hint” group (see S1 Table for a detailed explanation).

Immediately after each trial participants were asked to rate their anticipatory anxiety, pain intensity, and pain unpleasantness using computerised 11-point NRSs, which appeared as a number line on the laptop screen, with anchors of 0 (not at all anxious/no pain/not unpleasant), 5 (mildly anxious/mildly painful/mildly unpleasant), and 10 (extremely anxious/worst pain/extremely unpleasant) by pressing the corresponding number on a computer keypad with their finger. Keys 0–9 of the computer keypad had been coded in SuperLab to correspond to ratings of 0–9 (i.e., pressing the 0 key indicated a rating of 0), while the ‘+’ key of the computer keypad had been coded in SuperLab to correspond to a rating of 10. Stickers labelled with the numbers 0 to 10 had been placed over the relevant keys on the computer keypad for the sake of the participants. Participants were familiarised with the layout of the response keypad prior to commencing the pain anxiety task. Participants could only rate whole numbers (i.e., 7 or 8, but not 7.4). Throughout the pain anxiety task ratings were always presented and answered in the following order: anticipatory anxiety, pain intensity, and pain unpleasantness. The collection of subjective ratings of anticipatory anxiety, pain intensity, and pain unpleasantness at the conclusion of the trial rather than following the presentation of the visual cue) is consistent with previous research investigating the effects of cueing, fear, and threat on the anticipation and experience of pain [15, 16, 32, 33, 36, 37]. The difference between pain intensity and unpleasantness was illustrated by an analogy according to Price *et al*. [38]. In brief, the analogy relates the concepts of pain intensity and pain unpleasantness to listening to sound (e.g., a radio). As the volume of the radio increases, one can perceive how loud (i.e., intense) it sounds, as well as how unpleasant it is to hear it at that volume. This distinction is clarified because we know that pain unpleasantness is only partially related to stimulus intensity, and a range of other factors that influence affective experiences of pain. Participants were given up to 12 seconds to complete these ratings. Electrophysiological data (SCR and HR) were recorded continuously throughout the duration of the three phases of each individual trial (fixation, anticipation, and stimulus delivery).

### Mood questionnaires

The Beck Depression Inventory-II [BDI-II; 39] and the Beck Anxiety Inventory [BAI; 40] are both 21-item self-report measures designed to assess the severity of symptoms of depressive and anxious disorders within the last two weeks, respectively. These questionnaires have excellent psychometric properties and have been recommended for use in pain research [41]. Participants completed the BDI-II and BAI during the testing sessions prior to commencing the experimental task. Where responses from the BDI-II and/or BAI indicated that the participant was “at risk” of moderate anxious/depressive disorders and/or suicidal ideation (indicated by a score of ≥ eight on the BAI, a score of ≥ 19 on the BDI-II, and a score of ≥ two on item nine of the BDI-II, respectively) the testing session was stopped immediately, the participant was excluded from the study, and recommended to follow up with their doctor and/or the appropriate counselling services. Based on the BDI-II and BAI responses, seven individuals scored above the cut-off ranges. These potential participants were excluded due to the known associations between such disorders and altered pain perception [42].

### Procedure

Participants were assigned sequential participant numbers by author LMT. These participant numbers had previously been randomised to either the “Hint” or the “No Hint” groups by a research assistant who did not have participant contact and was not involved in data collection. Both the experimenter and participant were unaware of group allocation during the testing session. The experimenter was only unblinded to the participant’s group allocation after completing all data collection and extraction. Participants were informed that the current study was investigating how pain experience and physiological arousal varied over time.

Testing was always scheduled between 9am and 6:30pm, and all sessions followed the same general procedure. Participants were greeted by a male experimenter, who guided them through the experimental tasks. Participants completed baseline demographics and mood questionnaires (i.e., the BDI-II and BAI) before commencing the experiment. The current study formed part of a larger experimental investigation, and the results of other tasks administered have been discussed elsewhere [43].

### Data reduction and analysis

As per Bradley *et al*. [32], to assess the change in SCR and HR to the presentation of the visual cue (i.e., the anticipation period), change scores were generated for each half-second epoch following the cue presentation relative to the 0.5 s prior to cue onset (i.e., the last 0.5 s of the fixation period) for a period of 4 s. That is, each half second change score was calculated by subtracting the SCR and HR value 0.5 s prior to cue onset from the SCR and HR value from each half-second epoch that followed the onset of cue presentation. This resulted in eight change scores that indicated how SCR and HR changed from the pre-presentation baseline over time. The maximum change was then identified within a three second period during cue presentation as an index of maximal response during anticipation of the stimulus. The first second of the anticipatory period was excluded as HR and SCR responses to a stimulus will not show significant change until approximately one second after stimulus onset [44]. The same process was followed to determine electrophysiological responses during the stimulus period (except that these change scores remained relative to the last 0.5 s of the anticipation period) and the maximum change score was identified within the seven second period following the commencement of stimulus delivery from a total of fourteen 0.5 s epochs (again, the first second following the commencement of stimulus delivery was excluded from analysis). The change scores during anticipation and stimulus experience were screened for univariate outliers [45] and corrected using the Winsorizing method [46] within each participant group. The maximum SCR and HR change scores for each anticipation and stimulus response period were then averaged across the same trial types in each testing block. The anticipatory electrophysiological data and subjective ratings of anxiety were calculated in response to the presentation of each of the three non-ambiguous cues (i.e., high pain, low pain, and innocuous sensation) and the ambiguous cue (unknown pain). The electrophysiological data following stimulus delivery and the subjective ratings of pain intensity and pain unpleasantness were calculated in response to the intensity of the stimulus delivered (i.e., 45°C, 41°C, or 32°C) and the nature of the preceding cue (i.e., ambiguous or non-ambiguous).

Data were analysed with SPSS 20. A p value of < .05 was considered to be statistically significant, and Bonferroni corrections for multiple comparisons were applied to post hoc analysis results to counteract the likelihood of Type I error [47]. Demographic and questionnaire measures were compared between “Hint” and “No Hint” participant groups using t-tests (or Mann-Whitney U tests where violations of the assumption of normality were made). There were multiple violations of the assumption of normality for the subjective ratings data (particularly for ratings of the lower intensity stimuli), highlighting that careful consideration of the use of repeated measures analysis of variance (ANOVA) was required. We ran simple non-parametric comparisons, and ANOVAs, and found that the effects were consistent across approaches (S1 File), and so we elected to report the results from the ANOVAs. We also checked for the presence of within-subject outliers by comparing means with trimmed means (i.e., removing the lowest and highest value per variable within a subject). Comparison of the descriptive data, and results from repeated measures ANOVA of the trimmed and untrimmed means (S2 File) revealed the same results and so the untrimmed means are reported herein. Furthermore, ANOVA is not overly sensitive to violations of the assumption of normality, as has been found in simulation studies using a variety of non-normal distributions [48–50]. Subsequently, we were confident that the use of repeated measures ANOVA, for untrimmed means, was appropriate.

The effect of explicit cueing on anticipatory anxiety and arousal was determined with a mixed 2 x 3 x 4 repeated measures ANOVA with GROUP (Hint/No Hint) as the between-subjects factor, and BLOCK of the pain anxiety task (1/2/3) and colour of the CUE (Purple/Orange/Pink/Blue) as within-subjects factors. The effect of explicit cueing on the subjective response to the delivery of the stimulus was determined with a mixed 2 x 2 x 3 x 3 repeated measures ANOVA, with GROUP (Hint/No Hint) as the between-subjects factor, and NATURE (Non-ambiguous/Ambiguous), TEMPERATURE of the stimulus (45°C/41°C/32°C), and BLOCK of the pain anxiety task (1/2/3) as within-subject factors. Simple effects analyses were performed as post-hoc tests, where appropriate, with Bonferroni adjustments. Where Mauchly’s test indicated violations of the assumption of sphericity, the Huynh-Feldt correction was applied where estimates of sphericity were greater than 0.75, and the Greenhouse-Geisser correction was used when the estimates of sphericity were less than 0.75 [51].

Data from six participants was lost due to equipment malfunction (i.e., either the laptop computer controlling the Pathway unit or the laptop computer displaying the visual cues to the participants froze) on some trials. The final sample sizes were 46 for the HR data (25 males) and 47 for the SCR data (23 males). Despite the lost data the two participant groups (i.e., “Hint” and “No Hint”) retained approximately equal sample ratios for all analyses.

## Results

### Sample description

Participant groups did not differ with respect to age, depression, or anxiety (Table 2).

**Table 2:** Patient demographic data.

	Hint Group	No Hint Group	*p*
Sex (M:F)	13:13	13:13	---
Age	21.88 (3.49)	21.96 (3.61)	.897*
BDI-II	3.35 (3.38)	3.19 (3.24)	.824*
BAI-II	2.15 (2.09)	2.58 (1.86)	.269*

Data is presented as mean (SD).

* Denotes a violation of the assumption of normality, hence Mann Whitney *U* test performed.

### Subjective anxiety ratings

There was a main effect of GROUP on subjective anxiety ratings; *F*(1, 49) = 5.32, *p* = .025, partial eta squared (*η*^*2*^_*p*_) = .10. Participants in the “Hint” group reported higher levels of subjective anxiety (*M* = 2.61, SE = 0.36) compared to the “No Hint” participants (*M* = 1.38, SE = 0.36, *p* = .025). There was also a main effect of CUE on subjective anxiety ratings; *F*(1.82. 89.28) = 28.54, *p* < .001, *η*^*2*^_*p*_ = .37. The Purple cue (always preceding a 45°C thermal stimulus; *M* = 3.21, SE = 0.40) yielded higher ratings of subjective anxiety than the Orange (always preceding a 41°C thermal stimulus; *M* = 1.52, SE = 0.23, *p* < .001), Pink (always preceding a 32°C thermal stimulus; *M* = 1.12, SE = 0.23, *p* < .001), and Blue (always preceding a thermal stimulus of ambiguous temperature; *M* = 2.24, SE = 0.29, *p* < .001) cues. The Blue cue yielded higher ratings of subjective anxiety than the Orange (*p* = .007) and Pink (*p* = .001) cues. The Orange cue yielded higher ratings of subjective anxiety than the Pink cue (*p* = .02). There was no main effect of BLOCK on subjective anxiety ratings (*p* = .68).

**Fig 2:**
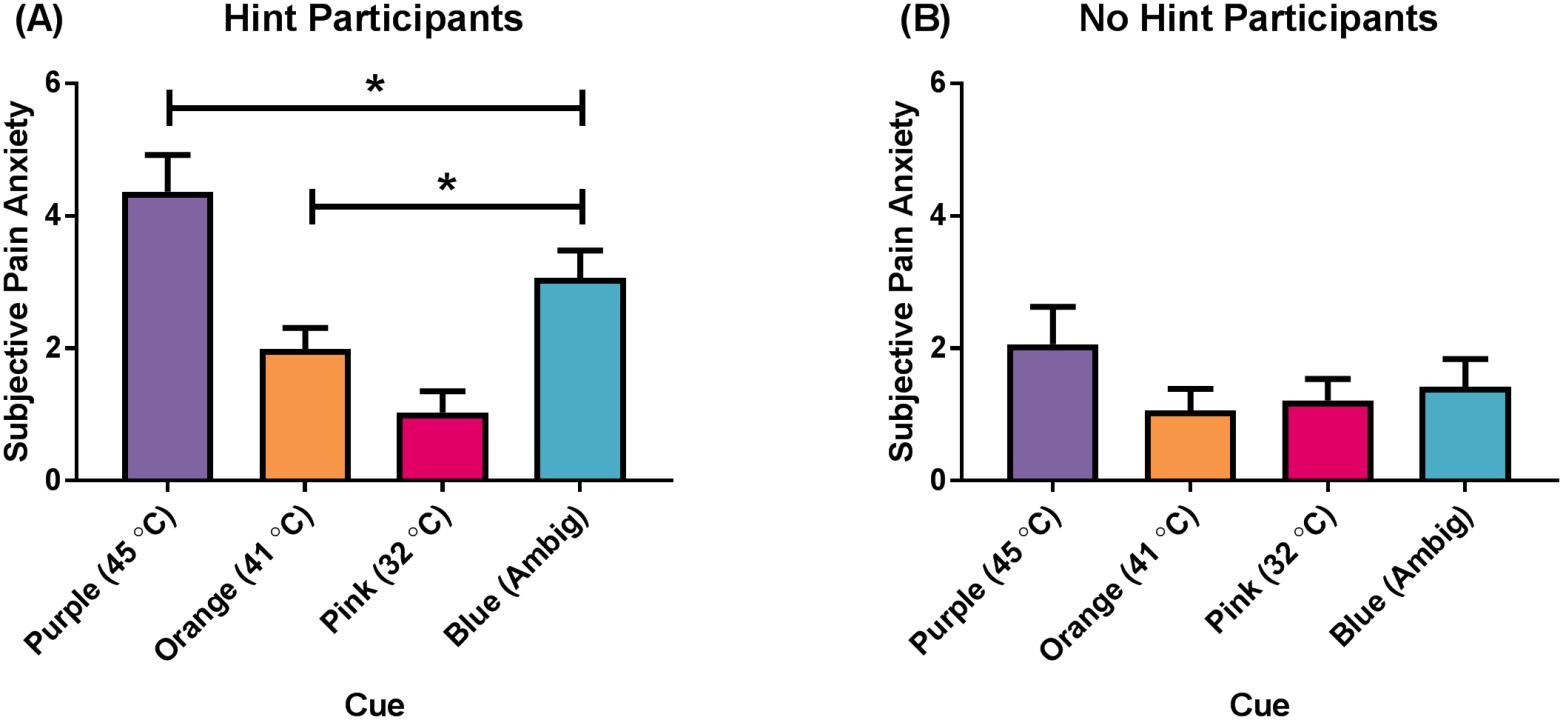
Subjective anxiety ratings (CUE x GROUP interaction). (A) “Hint” participants, and (B) “No Hint” participants. The Purple cue always preceded a 45°C stimulus, the Orange cue always preceded a 41°C stimulus, the Pink cue always preceded a 32°C stimulus, and the Blue cue always preceded an ambiguous stimulus. Data presented as mean (standard error). * *p* < .05.

There was a GROUP x CUE interaction; *F*(1.82, 89.28) = 9.60, *p* < .001, *η*^*2*^_*p*_ = .16; Fig 2. Post-hoc analyses revealed that in the “Hint” group, the Purple cue yielded higher ratings of subjective anxiety than the Orange (Mean difference = 2.38, *p* < .001), Pink (Mean difference = 3.34, *p* < .001), and Blue (Mean difference = 1.30, *p* = .001) cues; the Blue cue yielded higher ratings of subjective anxiety than the Orange (Mean difference = 1.08, *p* = .003) and Pink (Mean difference = 2.04, *p* = < .001) cues; and the Orange cue yielded higher ratings of subjective anxiety than the Pink cue (Mean difference = 0.96, *p* < .001). There were no differences in subjective anxiety ratings between cues for the “No Hint” group.

**Fig 3:**
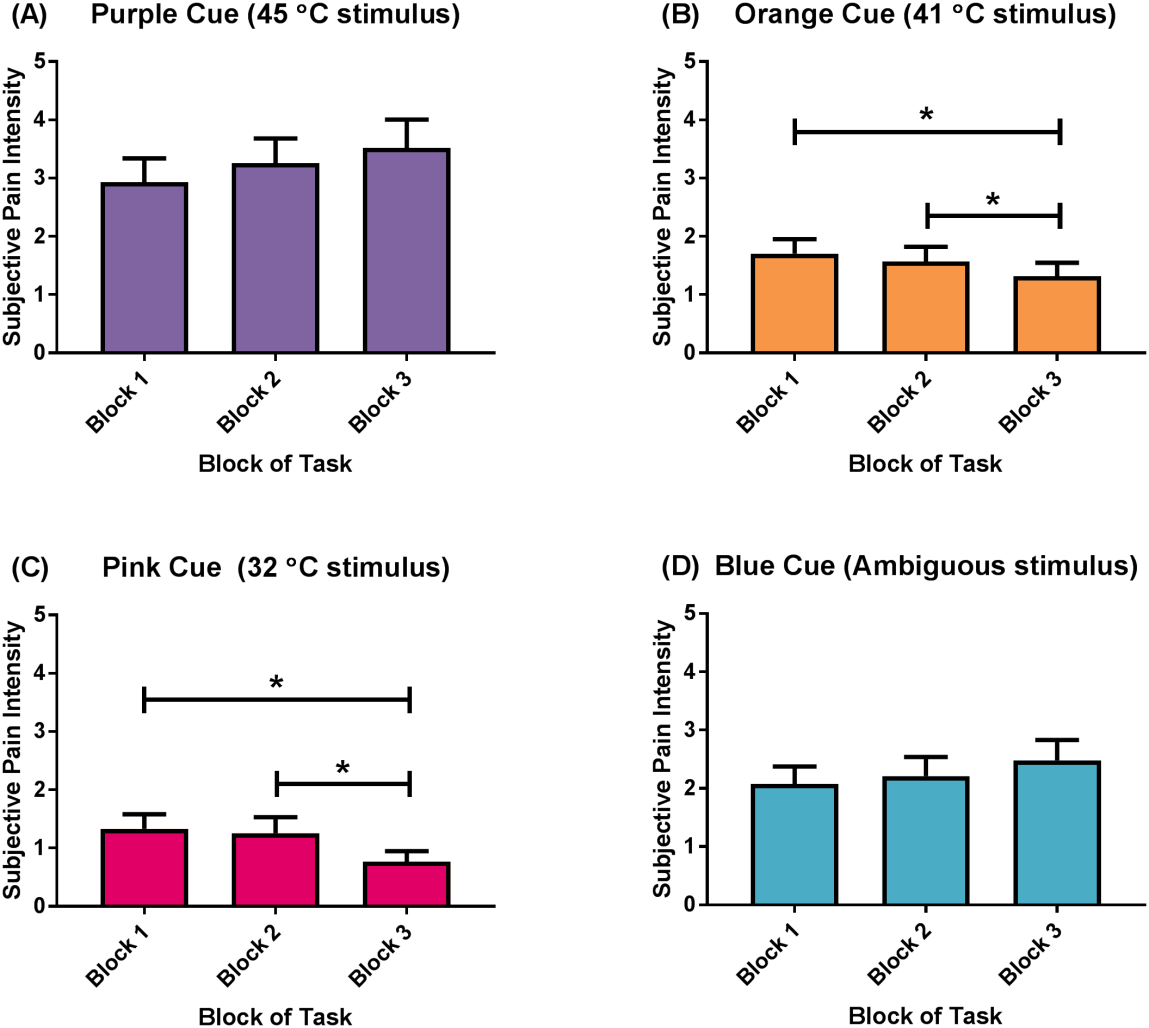
Subjective anxiety ratings (CUE x BLOCK) interaction. (A) Purple cue (always preceded a 45°C stimulus), (B) Orange cue (always preceded a 41°C stimulus), (C) Pink cue (always preceded a 32°C stimulus), and (D) Blue cue (always preceded a stimulus of ambiguous temperature). Data presented as mean (standard error). * *p* < .05.

There was a CUE x BLOCK interaction for subjective anxiety ratings; *F*(2.86, 140.30) = 8.27, *p* < .001, *η*^*2*^_*p*_ = .14; Fig 3. Post-hoc analyses revealed that this was specific to the Orange (i.e., the medium temperature of 41°C; Fig 3C) and Pink cues (i.e., the innocuous temperature of 32°C). The presentation of the Orange cue in Block 1 and Block 2 yielded higher ratings of subjective anxiety than the presentation of the same cue in Block 3 (Mean difference = 0.39, *p* = .01, Mean difference = .25, *p* = .04). Likewise, the presentation of the Pink cue in Block 1 and Block 2 yielded higher ratings of subjective anxiety than the presentation of the same cue in Block 3 (Mean difference = 0.56, *p* < .001, Mean difference = 0.49, *p* = .002, respectively). There were no differences in subjective ratings of anxiety over blocks in response to the Purple, or Blue cues. There were no other interactions (S2 Table).

### Anticipatory heart rate change

**Fig 4:**
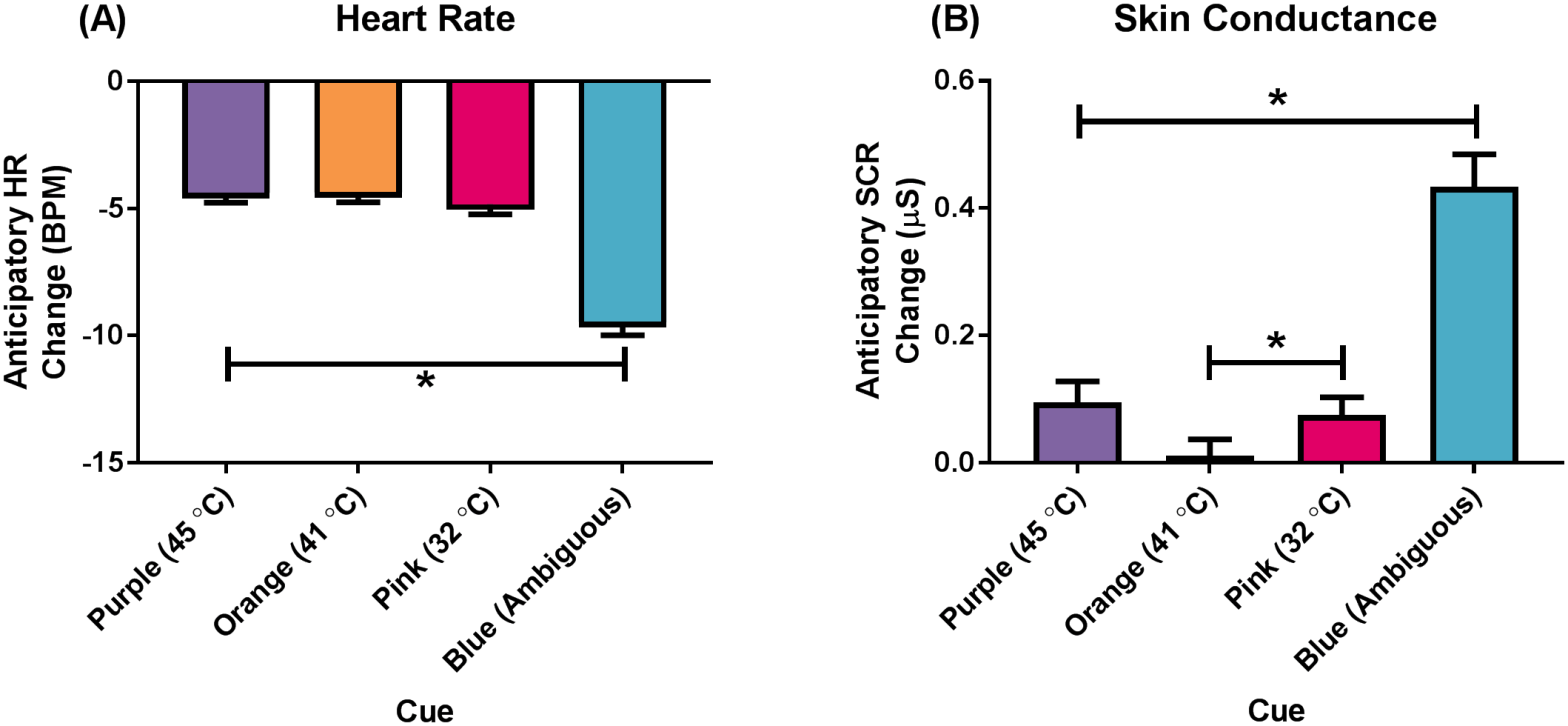
Anticipatory electrophysiological changes during cue presentation. (A) Heart rate change, and (B) Skin conductance response change. The Purple cue always preceded a 45°C stimulus, the Orange cue always preceded a 41°C stimulus, the Pink cue always preceded a 32°C stimulus, and the Blue cue always preceded an ambiguous stimulus. Data presented as mean (standard error). * *p* < .001.

There was no main effect of GROUP on anticipatory HR change (*p* = .16). There was a main effect of CUE on anticipatory HR change; *F*(3, 141) = 82.08, *p* < .001, *η*^*2*^_*p*_ = .64; Fig 4A. Post-hoc analyses revealed that the presentation of the Blue cue (always preceding a thermal stimulus of ambiguous temperature; *M* = -9.48, SE = 0.52) elicited a larger decrease in HR compared to the Purple (*M* = -4.38, SE = 0.39, *p* < .001), Orange (*M* = -4.35, SE = 0.40, *p* < .001), and Pink (*M* = -4.83, SE = 0.40, *p* < .001) cues. There was no main effect of BLOCK on anticipatory HR change (*p* = .14), nor were there any interaction effects (S3 Table).

### Anticipatory skin conductance response change

There was no main effect of GROUP on anticipatory SCR change (*p* = .88). There was a main effect of CUE on anticipatory SCR change; *F*(2.03, 95.25) = 48.34, *p* < .001, *η*^*2*^_*p*_ = .51; Fig 4B. Post-hoc analyses revealed that the presentation of the Blue cue (always preceding a thermal stimulus of ambiguous temperature; *M* = 0.43, SE = 0.05) elicited a larger SCR increase than the presentation of the Purple (*M* = 0.10, SE = 0.03, *p* < .001), Orange (*M* = 0.01, SE = 0.03, *p* < .001), and Pink (*M* = 0.08, SE = 0.03, *p* < .001) cues. The presentation of the Pink cue elicited a larger SCR increase than the presentation of the Orange cue (*p* = .01). There was no main effect of BLOCK on anticipatory SCR change (*p* = .58).

**Fig 5:**
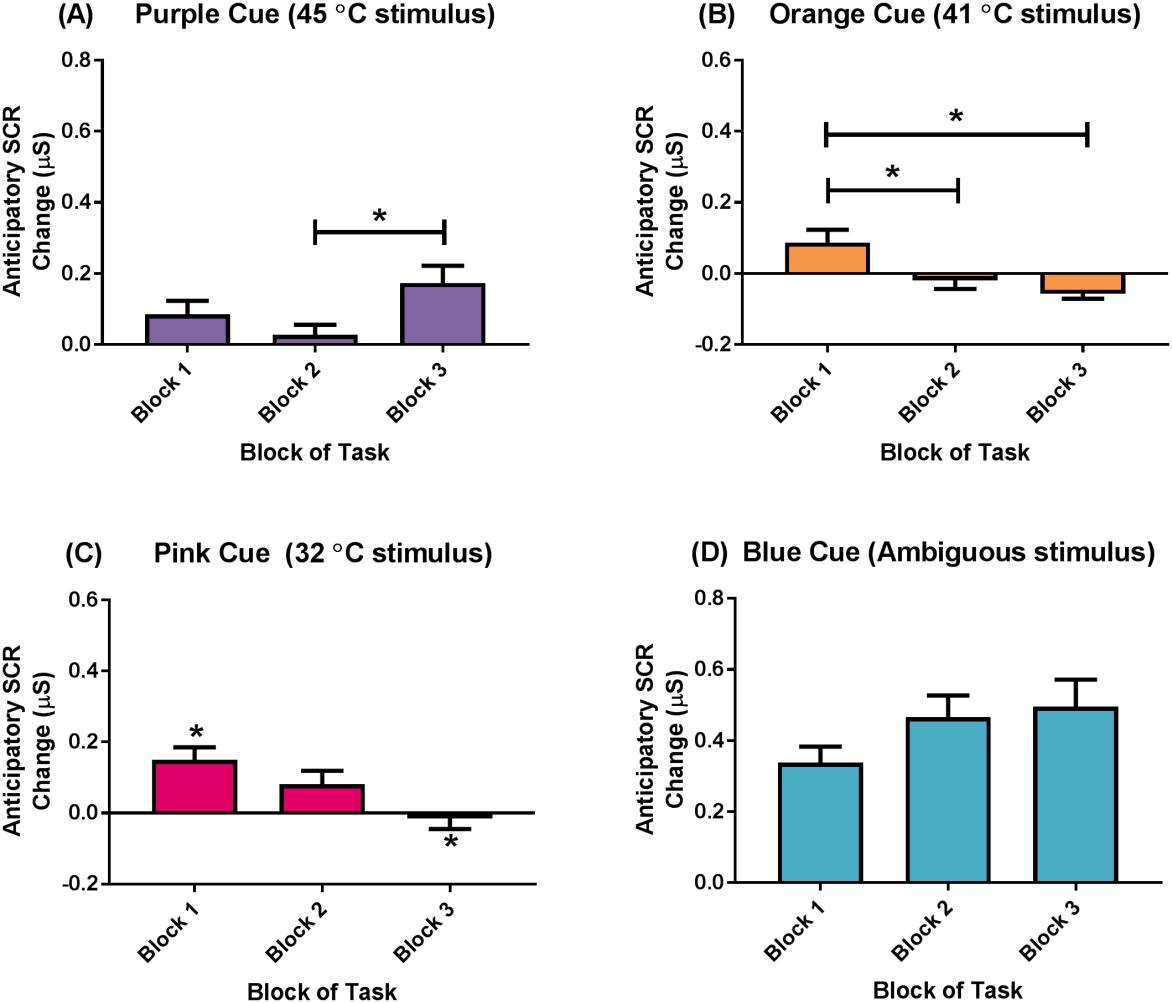
Anticipatory skin conductance response change (CUE x BLOCK) interaction. (A) Purple cue (always preceded a 45°C stimulus), (B) Orange cue (always preceded a 41°C stimulus), (C) Pink cue (always preceded a 32°C stimulus), and (D) Blue cue (always preceded a stimulus of ambiguous temperature). Data presented as mean (standard error). * *p* < .05.

There was a CUE x BLOCK interaction for anticipatory SCR change; *F*(6, 282) = 6.61, *p* < .001, *η*^*2*^_*p*_ = .12; Fig 5. Post-hoc analyses revealed that the presentation of the Purple cue (i.e., preceding the 45°C) in Block 3 elicited a larger SCR increase than the presentation of the same cue in Block 2 (Mean difference = 0.15, *p* = .002), but not Block 1. The presentation of the Orange cue (i.e., preceding the 41°C stimulus) in Block 1 elicited a larger SCR increase than the presentation of the same cue in Block 2 (Mean difference = 0.09, *p* = .04) and Block 3 (Mean difference = 0.13, *p* = .001). The presentation of the Pink cue (i.e., preceding innocuous 32°C stimulus) in Block 1 elicited a larger SCR increase than the presentation of the same cue in Block 3 (Mean difference = 0.15, *p* = .003), but not Block 2. There were no differences in the anticipatory SCR increase throughout the pain anxiety task in response to the presentation of the Blue cue. There were no other interaction effects (S4 Table).

### Subjective intensity ratings

There was no main effect of GROUP on pain intensity ratings (*p* = .82). There was a main effect of TEMPERATURE on pain intensity ratings; *F*(1.27, 62.02) = 275.71, *p* < .001, *η*^*2*^_*p*_ = .85. Post-hoc analyses revealed that the 45°C thermal stimuli (*M* = 6.16, SE = 0.34) were perceived as more intense than the 41°C (*M* = 2.56, SE = 0.21, *p* < .001) and 32°C (*M* = 0.31, SE = 0.07, *p* < .001) thermal stimuli. The 41°C thermal stimuli were perceived as more intense than the 32°C thermal stimuli (*p* < .001). There was a main effect of BLOCK on pain intensity ratings; *F*(1.59, 77.97) = 7.84, *p* = .002, *η*^*2*^_*p*_ = .14. Post-hoc analyse revealed that thermal stimuli delivered in Block 1 (*M* = 3.14, SE = 0.19) were perceived as more intense than thermal stimuli delivered in Block 3 (*M* = 2.87, SE = 0.19, *p* = .007), and thermal stimuli delivered in Block 2 (*M* = 3.02, SE = 0.18) were perceived as more intense than thermal stimuli delivered in Block 3 (*p* = .015). There was no main effect of NATURE on pain intensity ratings (i.e., thermal stimuli preceded by a non-ambiguous cue were perceived as similar to thermal stimuli preceded by an ambiguous cue; *p* = .26).

**Fig 6:**
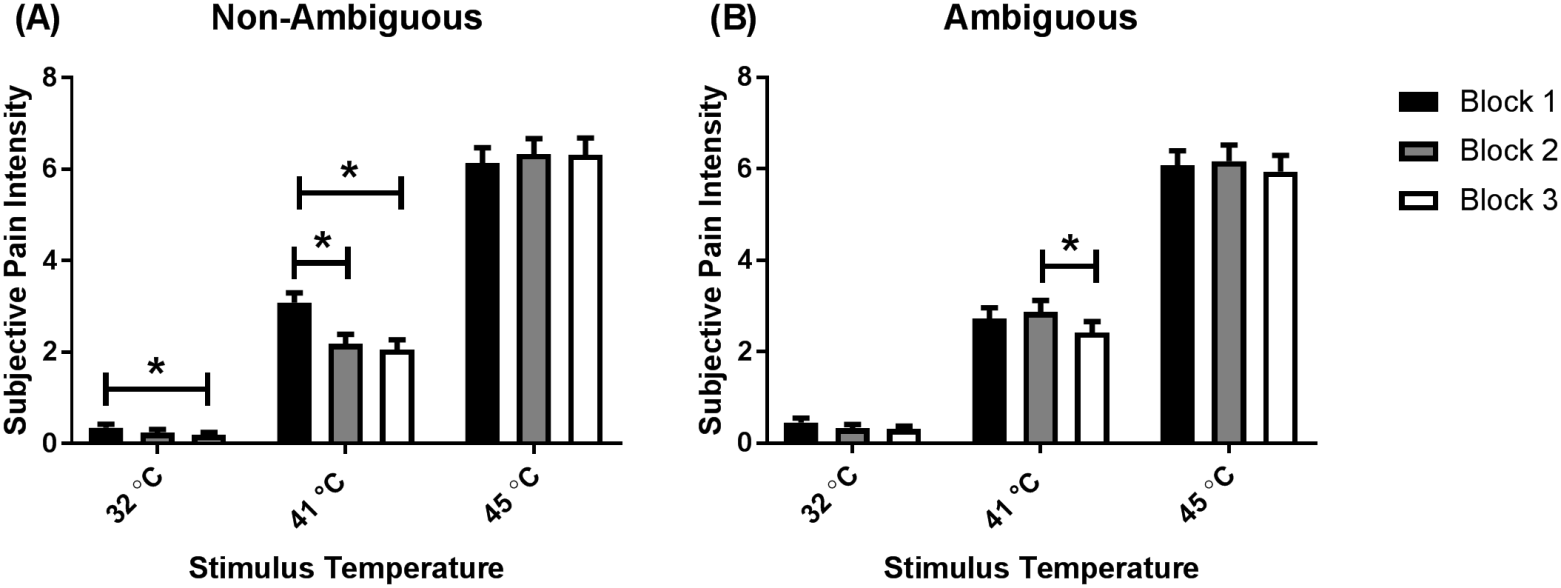
Subjective ratings of pain intensity (NATURE x TEMPERATURE x BLOCK). (A) Ratings for thermal stimuli preceded by non-ambiguous cues, and (B) ratings for thermal stimuli preceded by ambiguous cues. Ratings provided in Block 1 are represented by black bars, ratings provided in Block 2 are represented by grey bars, while ratings provided in Block 3 are represented by white bars. Data presented as mean (standard error). * *p* < .05.

There were NATURE x BLOCK (*F*(2, 98) = 9.08, *p* < .001, *η*^*2*^_*p*_ = .16), NATURE x TEMPERATURE (*F*(2, 98) = 11.04, *p* < .001, *η*^*2*^_*p*_ = .18), and TEMPERATURE x BLOCK (*F*(3.46, 169.75) = 8.21, *p* < .001, *η*^*2*^_*p*_ = .14) interactions. However, these two-way interactions were not probed due to the overarching three-way NATURE x TEMPERATURE x BLOCK interaction; *F*(3.48, 170.74) = 13.73, *p* < .001, *η*^*2*^_*p*_ = .22; Fig 6. Post-hoc analyses revealed that 41°C thermal stimuli preceded by a non-ambiguous cue in Block 1 were perceived as more intense than thermal stimuli of the same temperature preceded by a non-ambiguous cue in Block 2 (Mean difference = 0.90, *p* < .001) and Block 3 (Mean difference = 1.03, *p* < .001). The 32°C thermal stimuli preceded by a non-ambiguous cue in Block 1 were perceived as more intense than thermal stimuli of the same temperature preceded by a non-ambiguous cue in Block 3 (Mean difference = 0.16, *p* = .04). Finally, the 41°C thermal stimuli preceded by an ambiguous cue delivered in Block 2 were perceived as more intense than thermal stimuli of the same temperature preceded by an ambiguous cue delivered in Block 3 (Mean difference = 0.45, *p* = .01).

There was a GROUP x NATURE x TEMPERATURE interaction; *F*(2, 98) = 3.54, *p* = .03, *η*^*2*^_*p*_ = .07; S1 Fig. Post-hoc analyses revealed differences at each temperature for the “Hint” participants. The 45°C thermal stimuli preceded by a non-ambiguous cue (*M* = 6.38, SE = 0.47) were perceived as more intense than thermal stimuli of the same temperature preceded by an ambiguous cue (*M* = 5.90, SE = 0.47, *p* = .01). The 41°C thermal stimuli preceded by an ambiguous cue (*M* = 2.78, SE = 0.32) were perceived as more intense than thermal stimuli of the same temperature preceded by a non-ambiguous cue (*M* = 2.36, SE = 0.28, *p* = .001). The 32°C thermal stimuli preceded by an ambiguous cue (*M* = 0.38, SE = 0.11) were perceived as more intense than thermal stimuli of the same temperature preceded by a non-ambiguous cue (*M* = 0.24, SE = 0.09, *p* = .04). There were no differences in pain intensity ratings within the “No Hint” group. There were no other interaction effects (S5 Table).

### Subjective unpleasantness ratings

There was no main effect of GROUP on pain unpleasantness ratings (*p* = .99). There was a main effect of TEMPERATURE on pain unpleasantness ratings; *F*(1.22, 59.76) = 172.16, *p* < .001, *η*^*2*^_*p*_ = .78. Post-hoc analyses revealed that the 45°C thermal stimuli (*M* = 5.33, SE = 0.39) were perceived as more unpleasant than the 41°C (*M* = 1.52, SE = 0.19, *p* < .001) and 32°C (*M* = 0.13, SE = 0.04, *p* < .001) thermal stimuli. The 41°C thermal stimuli were perceived as more unpleasant than the 32°C thermal stimuli (*p* < .001). There was no main effect of NATURE (*p* = .44), or BLOCK (*p* = .08) on pain unpleasantness ratings.

**Fig 7:**
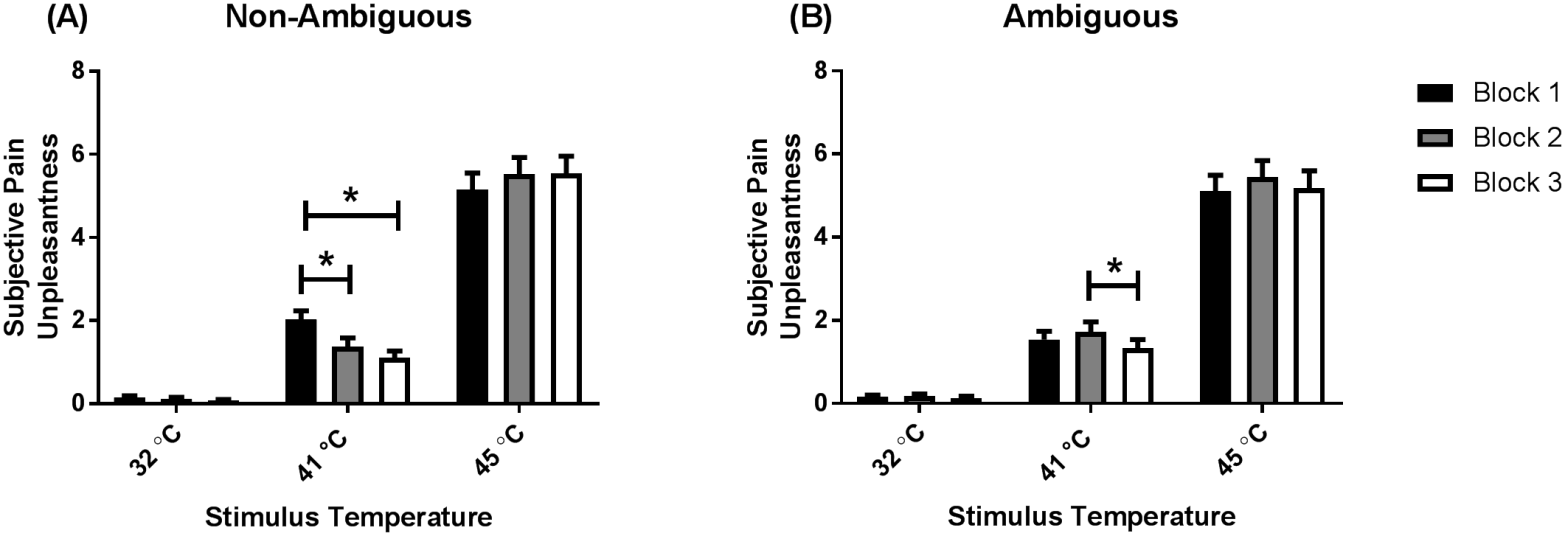
Subjective ratings of pain unpleasantness (NATURE x TEMPERATURE x BLOCK). (A) Ratings for thermal stimuli preceded by non-ambiguous cues, and (B) ratings for thermal stimuli preceded by ambiguous cues. Ratings provided in Block 1 are represented by black bars, ratings provided in Block 2 are represented by grey bars, while ratings provided in Block 3 are represented by white bars. Data presented as mean (standard error). * *p* < .05.

There were NATURE x BLOCK (*F*(1.72, 84.02) = 7.33, *p* = .002, *η*^*2*^_*p*_ = .13) and TEMPERATURE x BLOCK (*F*(2.73, 133.98) = 8.47, *p* < .001, *η*^*2*^_*p*_ = .15) interactions. However, these two-way interactions were not probed due to the overarching three-way NATURE x TEMPERATURE x BLOCK interaction; *F*(3.36, 164.85) = 10.45, *p* < .001, *η*^*2*^_*p*_ = .18, Fig 7. Post-hoc analyses revealed that for the 41°C thermal stimuli preceded by a non-ambiguous cue, stimuli delivered in Block 1 were perceived as more unpleasant than thermal stimuli of the same temperature delivered in Block 2 and Block 3 (Mean difference = 0.66, *p* < .001, Mean difference = 0.91, *p* = .01). Post-hoc analyses also revealed that the 41°C thermal stimuli preceded by an ambiguous cue in Block 2 were perceived as more unpleasant than thermal heat stimuli preceded by an ambiguous cue in Block 3 (Mean difference = 0.38, *p* = .046). There were no three-way interactions involving the 45°C and 32°C thermal stimuli. There were no other interaction effects (S6 Table).

### Stimulus heart rate change

There was no main effect of GROUP on stimulus HR change (*p* = .47). There was a main effect of NATURE on stimulus HR change; *F*(1, 47) = 4.16, *p* = .047, *η*^*2*^_*p*_ = .08. Post-hoc analyses revealed that thermal stimuli preceded by an ambiguous cue (*M* = -4.73, SE = 0.27) elicited a larger HR decrease than thermal stimuli preceded by a non-ambiguous cue (*M* = -4.36, SE = 0.32, *p* = .047). There was also a main effect of BLOCK on stimulus HR change; *F*(2, 94) = 8.03, *p* = .001, *η*^*2*^_*p*_ = .15. Post-hoc analyses revealed that thermal heat stimuli delivered in Block 2 (*M* = -4.76, SE = 0.29) and Block 3 (*M* = -4.80, SE = 0.34) elicited larger HR decreases than thermal heat stimuli delivered in Block 1 (*M* = -4.07, SE = 0.29; *p* = .009 and *p* = .001, respectively). There was no main effect of TEMPERATURE on stimulus HR change (*p* = .13).

There was a TEMPERATURE x BLOCK x GROUP interaction; *F*(3.46, 162.58) = 4.12, *p* = .005, *η*^*2*^_*p*_ = .08; S2 Fig. Post-hoc analyses revealed that these effects were specific to the “No Hint” participants, whereby the 45°C thermal stimuli delivered to “No Hint” participants in Block 2 (*M* = -6.11, SE = 0.63) elicited a larger HR decrease than 45°C thermal stimuli delivered to “No Hint” participants in Block 1 (*M* = -4.63, SE = 0.51, *p* = .01). In addition, the 32°C thermal stimuli delivered to “No Hint” participants in Block 3 (*M* = -5.28, SE = 0.58) elicited a larger HR decrease than 32°C thermal stimuli delivered to “No Hint” participants in Block 1 (*M* = -3.17, SE = 0.41, *p* = .001). There were no other differences in stimulus HR response, nor were there any other interaction effects (S7 Table).

### Stimulus skin conductance response change

There was no main effect of GROUP on stimulus SCR change (*p* = .12). There was a main effect of TEMPERATURE on stimulus SCR change; *F*(1.07, 50.43) = 41.52, *p* < .001, *η*^*2*^_*p*_ = .47. Post-hoc analyses revealed that the delivery of 45°C thermal stimuli (*M* = 1.01, SE = 0.14) elicited a larger SCR increase than the 41°C (*M* = 0.32, SE = 0.05, *p* < .001) and 32°C (*M* = 0.13, SE = 0.03, *p* < .001) thermal stimuli. The delivery of the 41°C thermal stimuli elicited a larger SCR increase than the delivery of the 32°C thermal stimuli (*p* < .001). There was a main effect of BLOCK on stimulus SCR change; *F*(1.65, 77.64) = 6.56, *p* = .004, *η*^*2*^_*p*_ = .12. Post-hoc analyses revealed that thermal stimuli delivered in Block 1 (*M* = 0.56, SE = 0.07) elicited larger SCR increases than the delivery of thermal stimuli in Block 2 (*M* = 0.47, SE = 0.07, *p* = .02) and Block 3 (*M* = 0.43, SE = 0.07, *p* = 0.02). There was no main effect of NATURE on stimulus SCR change (*p* = .88).

There was a NATURE x TEMPERATURE interaction; *F*(2, 94) = 4.09, *p* = .02, *η*^*2*^_*p*_ = .08. However, this two-way interaction was not probed due to the overarching three-way NATURE x TEMPERATURE x BLOCK interaction; *F*(3.47, 162.89) = 7.71, *p* < .001, *η*^*2*^_*p*_ = .14, S3 Fig. Post-hoc tests revealed that 41°C thermal stimuli preceded by a non-ambiguous cue delivered in Block 1 elicited a larger SCR increase than thermal stimuli of the same temperature preceded by a non-ambiguous cue delivered in Block 2 and Block 3 (Mean difference = 0.34, *p* < .001; Mean difference = 0.38, *p* < .001, respectively). The 32°C thermal stimuli preceded by a *non-ambiguous* cue delivered in Block 1 and Block 3 elicited larger SCR increases than thermal stimuli of the same temperature preceded by a non-ambiguous cue delivered in Block 2 (Mean difference = 0.22, *p* < .001; Mean difference = 0.17, *p* = .02, respectively). Finally, the 32°C thermal stimuli preceded by an *ambiguous* cue delivered in Block 1 and Block 2 elicited larger SCR increases than the same temperature stimuli preceded by an ambiguous cue delivered in Block 3 (Mean difference = 0.25, *p* < .001; Mean difference = 0.18, *p* < .01, respectively).

There was a TEMPERATURE x BLOCK x GROUP interaction; *F*(2.17, 102.16) = 3.97, *p* = .019, *η*^*2*^_*p*_ = .08; S4 Fig. Post-hoc analyses revealed that for the “Hint” group, the 41°C thermal stimuli delivered in Block 1 (*M* = 0.39, SE = 0.08) elicited a larger SCR increase than the same temperature stimuli delivered in Block 2 (*M* = 0.21, SE = 0.09, *p* = .01) and Block 3 (*M* = 0.13, SE = 0.08, *p* = 0.01). For the “Hint” group, the 32°C delivered in Block 1 (*M* = 0.18, SE = 0.06) elicited a larger SCR increase than the same temperature stimuli delivered in Block 2 (*M* = 0.04, SE = 0.04, *p* = .01). For the “No Hint” group, the 32°C thermal stimuli delivered in Block 1 (*M* = 0.29, SE = 0.06) elicited a larger SCR increase than the same stimuli delivered in Block 3 (*M* = 0.10, SE = 0.06, *p* = .001). There were no other interaction effects (S8 Table).

## Discussion

The present study investigated the effects of explicit cueing, and the uncertain nature of specific cues, on anticipatory anxiety and arousal when experiencing repeated painful thermal stimulations, and whether these effects changed over the task. The results showed that the effects of explicit cueing (i.e., being forewarned of the relationship between a visual cue and its paired thermal stimulus) were limited to subjective anticipatory anxiety. There was no overall effect of explicit cueing on the perceived intensity and unpleasantness of the thermal heat stimuli. Furthermore, there was no effect of explicit cueing on electrophysiological arousal in anticipation of, or in response to, the delivery of a thermal heat stimulus.

The finding that explicit cueing enhanced anticipatory anxiety is in line with previous research, where priming patients with expectations of pain prior to a potentially painful event results in greater levels of self-reported anxiety [52]. Participants who had a priori knowledge of the cue-stimulus pairings (i.e., the “Hint” group) reported higher anxiety in response to the presentation of the Purple (preceding the 45°C stimulus) cue compared to the Blue (ambiguous), Orange (41°C), and Pink (32°C) cues. However, the participants who did not have a priori knowledge of the cue-stimulus pairings (i.e., the “No Hint” group) did not report different levels of subjective anxiety in response to the different cues, and had lower anticipatory anxiety overall compared to the “Hint” group. Therefore, being aware of the meaning of the relationship between a cue and stimulus in an experimental situation increased anticipatory anxiety.

The current study did not replicate previously reported findings of explicit cueing on anticipatory electrophysiological responses [14, 21, 32], subjective ratings of pain intensity and unpleasantness [30, 31], and the electrophysiological responses during the delivery of the stimulus. Previous studies propose that being aware of a potentially painful stimulus induces activity in the amygdala that potentiates defensive startle reflexes in preparation for defensive action [53–55]. The amygdala activation also elicits activity in the sweat glands, increasing the SCR, leads to cardiovascular activity that elicits HR deceleration, and increases sensitivity to acute pain [14, 21, 30–32, 56]. The lack of a main effect of explicit cueing on the electrophysiological anticipation and subjective experience of pain suggests that the manipulation of explicit cueing in the current study was essentially non-apparent.

Despite the limited effects of explicit cueing on the anticipation and experience of pain, there were widespread effects of the nature of the cue presented prior to stimulus delivery. These effects were observed to vary throughout the duration of the task, whereby subjective ratings of anticipatory anxiety, pain intensity, and pain unpleasantness, and electrophysiological responses changed across blocks. These findings may be partly explained by a modification of the *interoceptive predictive coding model* [57–59]. Predictive coding refers to the phenomenon whereby the brain continually produces models of the surrounding environment based on information that is currently available in the environment and memories of previous similar environments, to predict or anticipate sensory input [60]. Put more simply, the brain dictates how we perceive, and react to, internal and external nociceptive sensations based on all currently available information in the surrounding environment, and from past experiences and memories [3, 61].

There were no effects of explicit cueing on the anticipatory electrophysiological responses, but there were differences in the change in HR and SCR depending on the colour of the presented cue. The presentation of the *blue* cue (preceding stimuli of an ambiguous temperature) resulted in larger decreases in HR compared to the other visual cues. Likewise, the anticipatory SCR increase was larger in response to the *blue* cue compared to the other visual cues. Collectively these results are consistent with previous findings showing increased electrophysiological activity during the presentation of a cue that threatens pain [16, 21, 32]. The enhanced anticipatory electrophysiological responses to the ambiguous cue can be interpreted in terms of predictability and uncertainty, in line with the interoceptive predictive coding model. The presentation of the blue cue was associated with uncertainty, as the following thermal stimulus could be any of the three stimulus temperatures, one of which was quite painful. In contrast, the presentation of the remaining three cues could be associated with a predictable thermal stimulus, as the purple, orange, and pink cues always preceded 45°C, 41°C, and 32°C thermal stimuli, respectively. The uncertainty associated with the blue cue may have resulted in neurophysiological activity (possibly via the amygdala), leading to increased SCR and greater HR deceleration when anticipating the stimulus [14, 32]. The current findings suggest that through interoceptive predictive coding, as participants progress through the task and become more familiar with the relationship between the cues and the thermal stimuli they become less anxious about (and reactive to) cues that are associated with little or no pain, and more anxious about cues that are (or might be) associated with more intense pain.

There were a number of changes in subjective and electrophysiological responses over time (i.e., across blocks) for the pain anxiety task. As the task progressed, participants became less anxious (i.e., they reported lower anticipatory anxiety and displayed decreased electrophysiological arousal) when presented with cues that were paired with a lower temperature thermal stimulus (i.e., the Orange and Pink cues). In contrast, participants appeared to become more anxious when presented with the cue that was paired with a higher temperature thermal stimulus (i.e., the Purple cue), or with a cue that was paired with a thermal stimulus of ambiguous temperature (i.e., the Blue cue). Again, these results can be interpreted via the predictive coding model, where the subjective and electrophysiological anticipatory responses to the visual cues change throughout the task as a result of participants becoming more familiar with the predictability and the uncertainty of the cues. Participants reported a decrease in the perceived intensity and unpleasantness of the thermal stimuli, particularly for the 41°C thermal stimuli (and not the 45°C or 32°C stimuli), but displayed an increase in electrophysiological arousal as the task progressed.

The ambiguous cue appeared to impact the perception of the intensity of the moderate (41°C) and high (45°C) temperature stimuli differently. For higher temperature stimuli, an ambiguous cue increased subjective intensity, but for the lower temperatures, it decreased perceived intensity. This finding is consistent with previous work on the divergent effects of fear (an immediate alarm reaction to a present threat, characterised by impulses to escape) and anxiety (a future-oriented emotion characterised by negative affect and apprehensive anticipation of potential threats) on pain perception performed by Rhudy et al. [56]. In the study by Rhudy et al., fear resulted in a decrease in pain sensitivity, whereas anxiety lead to an increase in pain sensitivity. The findings of the current study suggest that (for the stimuli of a lower temperature) the presentation of the ambiguous cue enhanced anticipatory anxiety about the stimulus, and increased sensitivity to the subsequent stimulus. This effect was seen only for the subjective intensity of the stimulus, but not the perceived unpleasantness. The absence of such an interaction for the subjective ratings of pain unpleasantness was interesting, yet consistent with the fact that we know that pain unpleasantness is only partially related to stimulus intensity, and that there are a range of other factors that influence affective experiences of pain, such as emotional arousal [62].

There were divergent electrophysiological responses to the delivery of the thermal heat stimuli across the blocks of the pain anxiety task. The thermal stimuli delivered in Block 1 elicited the smallest HR deceleration, compared to Block 2 and Block 3, but thermal stimuli delivered in Block 1 elicited the largest SCR increase in Block 1, compared to Block 2 and Block 3. That is, the magnitude of HR deceleration to the thermal stimulus increased as the task progressed, but the increase in SCR to the thermal stimulus decreased as the task progressed. The progressive decrease in the SCR to stimulus delivery throughout the blocks of the pain anxiety task suggests that participants became desensitised, or habituated, to the thermal heat stimuli. Consequently, there may be a reduction of the sympathetic nervous system response to the thermal heat stimuli as the task progresses, but no such reduction for the parasympathetic nervous system. This habituation was also seen in the subjective ratings of pain intensity, where the ratings in the third block of the task were significantly lower than the ratings in the first two blocks. The progressive increase in HR deceleration to thermal stimuli throughout the experiment was unexpected, however, for two reasons. First, previous research has reported that (after an initial reduction) HR should rise as participants are exposed to experimentally-induced pain [9]. Second, we would expect the HR deceleration to become less pronounced if habituation was occurring. One possible explanation for these unexpected findings is that there may be a surge of inhibitory parasympathetic activity following the delivery of the stimulus (and the level of activity in the parasympathetic nervous system increases as the task progresses) to re-establish homeostatic balance, which further decreases HR [63].

### Strengths and limitations

A key strength of this study was that both the experimenter and participants were rigorously blinded to the group allocation. It may be possible that previous empirical studies that did not follow such blinding may have introduced potential biases. A second strength was the use of various stimulus temperatures within the task (i.e., 45°C, 41°C, and 32°C), rather than simply a painful and non-painful stimulus option. Selecting a range of stimulus temperatures (designed to cover a range of responses from not at all painful through to moderately painful) allowed us to examine the intricacies in the subjective experience of pain across a larger stimulus-response range. Finally, in accordance with our experimental design, the temperatures utilised in this study were sufficiently different from each other (i.e., 45°C stimuli perceived as more intense and unpleasant than the 41°C and 32°C; 41°C stimuli perceive as more intense and unpleasant than 32°C stimuli).

Several limitations of this study should be noted. First, the participants were young, healthy participants who did not have any current or prior medical or psychological condition that would increase sensitivity to pain. The results might therefore not generalise to older persons, or to clinical (i.e., a chronic pain) populations, who may display altered subjective and physiological responses when exposed to a potentially painful stimulus or event. Second, the findings may not generalise to other forms of noxious somatosensory stimulation (e.g., mechanical, chemical etc.) as we only used thermal heat stimuli. Third, participants were unable to provide ratings of subjective anxiety, pain intensity, and pain unpleasantness until the end of the four second stimulus delivery period. While we clearly instructed participants to indicate their level of anxiety at the time *prior to the onset of the stimulus*, the ratings may have been affected by the stimulus intensity. Nonetheless, the retrospective rating of anticipatory anxiety may have been impacted by the subsequent temperature and perceiving intensity or unpleasantness of the thermal stimulus. It is possible that participants did not rate each individual stimulus as a unique entity, rather providing the same subjective ratings in response to the visual cues and stimulus intensities regardless of the stage of the experimental task. However, the collection of ratings at the conclusion of the trial or the task, rather than following the presentation of the visual cue, is consistent with previous research investigating the effects of cueing, fear, and threat on the anticipation and experience of pain [15, 16, 32, 33, 36, 37].

Fourth, the thermal heat stimulus temperatures applied were consistent for all participants (i.e., all participants received stimulations of 45°C, 41°C, and 32°C). It is possible that if the stimulus temperatures utilised in this experiment were personalised to individual pain sensitivities then explicit cueing may have had a larger effect, which may explain the absence of an effect of explicit cueing on the perceived intensity and unpleasantness of the stimuli. It is important to note, however, that previous studies investigating fear and threat on the anticipation and experience of pain have utilised standardised stimulus temperatures and intensities [32, 33, 36]. Finally, we did not specifically ask participants whether they had identified the relationship between the colour of the visual cue and the temperature of the following thermal stimulus. However, nine of the 26 participants in the “No Hint” group (35%) freely commented during debriefing at the end of the session that they had either learned, or had become suspicious of, the relationship between the colour of the visual cue and the temperature of the following thermal stimulus at some point throughout the pain anxiety task. With respect to the implicit learning literature, a free report of 35% is relatively high [64]. Had we specifically ascertained this information at the end of the testing session (e.g., through a simple questionnaire) then additional informative analyses could have been performed to further characterise learning effects. The use of a questionnaire, or other measure of awareness of the association may have provided more conclusive evidence about how many participants in the “No Hint” group had become aware of the association between the colour of the cue and the temperature of the paired thermal stimulus [64].

### Conclusions

Our results offer evidence of a subtle yet significant effect of explicit cueing on anticipatory anxiety prior to the delivery of a painful thermal stimulus. More robust effects of ambiguous cueing were observed, whereby painful stimuli preceded by ambiguous and non-ambiguous cues were perceived at different intensities, depending on the temperature of the stimulus. Predictive coding of experimentally-induced painful stimuli influences the subjective and electrophysiological anticipation and experience of the stimuli. Further studies are now required to identify the influence of subjective anxiety on the physiological response to the anticipation and delivery of repeated painful thermal stimulations in older and clinical (e.g., chronic pain) populations, and to further examine the varying nature of subjective responses to a mildly painful thermal heat stimulus.

## Supporting information

S1 Fig Subjective ratings of pain intensity (NATURE x TEMPERATURE x GROUP). (A) “Hint” group, and (B) “No Hint” group. Stimuli preceded by non-ambiguous cues are represented by black bars, whereas stimuli preceded by ambiguous cues are represented by grey bars. Data presented as mean (standard error). * *p* < .008.
(TIF)

S2 Fig Heart rate change to stimulus delivery (GROUP x BLOCK x TEMPERATURE). (A) “Hint” group, and (B) “No Hint” group. HR changes to thermal stimuli delivered in Block 1 are represented by the black bars, changes to thermal stimuli delivered in Block 2 are represented by the grey bars, while changes to thermal stimuli delivered in block 3 are represented by the white bars. Data presented as mean (standard error). * *p* < .002.
(TIF)

S3 Fig Skin conductance response changes to stimulus delivery (NATURE x TEMPERATURE x BLOCK). (A) SCR changes for thermal stimuli preceded by non-ambiguous cues, and (B) SCR changes for thermal stimuli preceded by ambiguous cues. Stimuli delivered in Block 1 are represented by black bars, stimuli delivered in Block 2 are represented by grey bars, while stimuli delivered in Block 3 are represented by white bars. Data presented as mean (standard error). * *p* < .05.
(TIF)

S4 Fig Skin conductance response changes to stimulus delivery (GROUP x BLOCK x TEMPERATURE). (A) “Hint” group, and (B) “No Hint” group. SCR changes to thermal stimuli delivered in Block 1 are represented by the black bars, changes to thermal stimuli delivered in Block 2 are represented by the grey bars, while changes to thermal stimuli delivered in Block 3 are represented by the white bars. Data presented as mean (standard error). * *p* < .002.
(TIF)

S1 Table Instructions provided to participants. (DOCX)

S2 Table Summary of main and interaction effects for subjective anxiety ratings. (DOCX)

S3 Table Summary of main and interaction effects for anticipatory heart rate response. (DOCX)

S4 Table Summary of main and interaction effects for anticipatory skin conductance response. (DOCX)

S5 Table Summary of main and interaction effects for pain intensity ratings. (DOCX)

S6 Table Summary of main and interaction effects for pain unpleasantness ratings. (DOCX)

S7 Table Summary of main and interaction effects for stimulus heart rate response. (DOCX)

S8 Table Summary of main and interaction effects for stimulus skin conductance response. (DOCX)

S1 File Evidence for consistent effects across non-parametric comparisons. Summary of non-parametric statistical analyses.
(DOCX)

S2 File Evidence for consistent effects when using trimmed means. Summary of statistical analyses using when trimmed means.
(DOCX)

## References

[1] de C Williams AC, Craig KD. Updating the definition of pain. Pain. 2016;157(11):2420–3. doi: 10.1097/j.pain.0000000000000613 27200490 10.1097/j.pain.0000000000000613

[2] Wiech K, Tracey I. The influence of negative emotions on pain: Behavioral effects and neural mechanisms. Neuroimage. 2009;47(3):987–94. doi: 10.1016/j.neuroimage.2009.05.059 19481610 10.1016/j.neuroimage.2009.05.059

[3] Tsay A, Allen TJ, Proske U, Giummarra MJ. Sensing the body in chronic pain: A review of psychophysical studies implicating altered body representation. Neuroscience and biobehavioral reviews. 2015;52:221–32. Epub 2015/03/19. doi: 10.1016/j.neubiorev.2015.03.004 . 25783221 10.1016/j.neubiorev.2015.03.004

[4] Loggia ML, Juneau M, Bushnell MC. Autonomic responses to heat pain: Heart rate, skin conductance, and their relation to verbal ratings and stimulus intensity. Pain. 2011;152(3):592–8. doi: 10.1016/j.pain.2010.11.032 21215519 10.1016/j.pain.2010.11.032

[5] Hampf G. Influence of cold pain in the hand on skin impedance, heart rate and skin temperature. Physiology & behavior. 1990;47(1):217–8. Epub 1990/01/01. . 2326340 10.1016/0031-9384(90)90064-b

[6] Kregel KC, Seals DR, Callister R. Sympathetic nervous system activity during skin cooling in humans: relationship to stimulus intensity and pain sensation. The Journal of physiology. 1992;454:359–71. Epub 1992/08/01. ; 1474495 10.1113/jphysiol.1992.sp019268 PMC1175609

[7] Lavigne GJ, Zucconi M, Castronovo V, Manzini C, Veglia F, Smirne S, et al Heart rate changes during sleep in response to experimental thermal (nociceptive) stimulations in healthy subjects. Clinical neurophysiology: official journal of the International Federation of Clinical Neurophysiology. 2001;112(3):532–5. Epub 2001/02/27. . 11222976 10.1016/s1388-2457(00)00558-7

[8] Moltner A, Holzl R, Strian F. Heart rate changes as an autonomic component of the pain response. Pain. 1990;43(1):81–9. Epub 1990/10/01. . 2277719 10.1016/0304-3959(90)90052-F

[9] Tousignant-Laflamme Y, Rainville P, Marchand S. Establishing a link between heart rate and pain in healthy subjects: a gender effect. The journal of pain: official journal of the American Pain Society. 2005;6(6):341–7. Epub 2005/06/10. doi: 10.1016/j.jpain.2005.01.351 . 15943955 10.1016/j.jpain.2005.01.351

[10] Dube AA, Duquette M, Roy M, Lepore F, Duncan G, Rainville P. Brain activity associated with the electrodermal reactivity to acute heat pain. Neuroimage. 2009;45(1):169–80. Epub 2008/11/26. doi: 10.1016/j.neuroimage.2008.10.024 . 19027077 10.1016/j.neuroimage.2008.10.024

[11] Eriksson M, Storm H, Fremming A, Schollin J. Skin conductance compared to a combined behavioural and physiological pain measure in newborn infants. Acta paediatrica (Oslo, Norway: 1992). 2008;97(1):27–30. Epub 2007/12/07. doi: 10.1111/j.1651-2227.2007.00586.x . 18052991 10.1111/j.1651-2227.2007.00586.x

[12] Harrison D, Boyce S, Loughnan P, Dargaville P, Storm H, Johnston L. Skin conductance as a measure of pain and stress in hospitalised infants. Early human development. 2006;82(9):603–8. Epub 2006/03/02. doi: 10.1016/j.earlhumdev.2005.12.008 . 16507342 10.1016/j.earlhumdev.2005.12.008

[13] Schestatsky P, Valls-Sole J, Costa J, Leon L, Veciana M, Chaves ML. Skin autonomic reactivity to thermoalgesic stimuli. Clinical autonomic research: official journal of the Clinical Autonomic Research Society. 2007;17(6):349–55. Epub 2007/12/01. doi: 10.1007/s10286-007-0446-8 . 18049833 10.1007/s10286-007-0446-8

[14] Seifert F, Schuberth N, De Col R, Peltz E, Nickel FT, Maihofner C. Brain activity during sympathetic response in anticipation and experience of pain. Human brain mapping. 2013;34(8):1768–82. Epub 2012/03/23. doi: 10.1002/hbm.22035 . 22438199 10.1002/hbm.22035 PMC6870103

[15] Colloca L, Benedetti F, Pollo A. Repeatability of autonomic responses to pain anticipation and pain stimulation. European journal of pain (London, England). 2006;10(7):659–65. Epub 2005/12/13. doi: 10.1016/j.ejpain.2005.10.009 . 16337150 10.1016/j.ejpain.2005.10.009

[16] Ploghaus A, Narain C, Beckmann CF, Clare S, Bantick S, Wise R, et al Exacerbation of pain by anxiety is associated with activity in a hippocampal network. The Journal of neuroscience: the official journal of the Society for Neuroscience. 2001;21(24):9896–903. Epub 2001/12/12. . 11739597 10.1523/JNEUROSCI.21-24-09896.2001 PMC6763058

[17] Barlow DH, Chorpita BF, Turovsky J, editors. Fear, panic, anxiety, and disorders of emotion. Nebraska Symposium on Motivation; 1996: Current theory in research and motivation. 8912311

[18] Oka S, Chapman CR, Kim B, Nakajima I, Shimizu O, Oi Y. Pupil dilation response to noxious stimulation: effect of varying nitrous oxide concentration. Clinical neurophysiology: official journal of the International Federation of Clinical Neurophysiology. 2007;118(9):2016–24. Epub 2007/07/25. doi: 10.1016/j.clinph.2007.04.023 . 17646133 10.1016/j.clinph.2007.04.023

[19] Tousignant-Laflamme Y, Marchand S. Sex differences in cardiac and autonomic response to clinical and experimental pain in LBP patients. European journal of pain (London, England). 2006;10(7):603–14. Epub 2005/11/22. doi: 10.1016/j.ejpain.2005.09.003 . 16298532 10.1016/j.ejpain.2005.09.003

[20] Yang LL, Niemann CU, Larson MD. Mechanism of pupillary reflex dilation in awake volunteers and in organ donors. Anesthesiology. 2003;99(6):1281–6. Epub 2003/11/26. . 14639139 10.1097/00000542-200312000-00008

[21] Bradley MM, Moulder B, Lang PJ. When good things go bad: the reflex physiology of defense. Psychological science. 2005;16(6):468–73. Epub 2005/06/10. doi: 10.1111/j.0956-7976.2005.01558.x . 15943673 10.1111/j.0956-7976.2005.01558.x

[22] Keltner JR, Furst A, Fan C, Redfern R, Inglis B, Fields HL. Isolating the modulatory effect of expectation on pain transmission: a functional magnetic resonance imaging study. The Journal of neuroscience: the official journal of the Society for Neuroscience. 2006;26(16):4437–43. Epub 2006/04/21. doi: 10.1523/jneurosci.4463-05.2006 . 16624963 10.1523/JNEUROSCI.4463-05.2006 PMC6674009

[23] Petrovic P, Ingvar M. Imaging cognitive modulation of pain processing. Pain. 2002;95(1–2):1–5. Epub 2002/01/16. . 11790461 10.1016/s0304-3959(01)00467-5

[24] Ploghaus A, Tracey I, Gati JS, Clare S, Menon RS, Matthews PM, et al Dissociating pain from its anticipation in the human brain. Science (New York, NY). 1999;284(5422):1979–81. Epub 1999/06/18. . 10373114 10.1126/science.284.5422.1979

[25] Price DD, Milling LS, Kirsch I, Duff A, Montgomery GH, Nicholls SS. An analysis of factors that contribute to the magnitude of placebo analgesia in an experimental paradigm. Pain. 1999;83(2):147–56. Epub 1999/10/27. . 10534585 10.1016/s0304-3959(99)00081-0

[26] Wager TD. Expectations and anxiety as mediators of placebo effects in pain. Pain. 2005;115(3):225–6. Epub 2005/05/25. doi: 10.1016/j.pain.2005.03.018 . 15911149 10.1016/j.pain.2005.03.018

[27] Wager TD, Rilling JK, Smith EE, Sokolik A, Casey KL, Davidson RJ, et al Placebo-induced changes in FMRI in the anticipation and experience of pain. Science (New York, NY). 2004;303(5661):1162–7. Epub 2004/02/21. doi: 10.1126/science.1093065 . 14976306 10.1126/science.1093065

[28] Crombez G, Eccleston C, Baeyens F, Eelen P. Attentional disruption is enhanced by the threat of pain. Behaviour research and therapy. 1998;36(2):195–204. Epub 1998/06/05. . 9613025 10.1016/s0005-7967(97)10008-0

[29] Crombez G, Eccleston C, Baeyens F, Eelen P. When somatic information threatens, catastrophic thinking enhances attentional interference. Pain. 1998;75(2–3):187–98. Epub 1998/05/16. . 9583754 10.1016/s0304-3959(97)00219-4

[30] Ott J, Aust S, Nouri K, Promberger R. An everyday phrase may harm your patients: the influence of negative words on pain during venous blood sampling. The Clinical journal of pain. 2012;28(4):324–8. Epub 2011/10/18. doi: 10.1097/AJP.0b013e3182321cc3 . 22001664 10.1097/AJP.0b013e3182321cc3

[31] Dutt-Gupta J, Bown T, Cyna AM. Effect of communication on pain during intravenous cannulation: a randomized controlled trial. British journal of anaesthesia. 2007;99(6):871–5. Epub 2007/11/06. doi: 10.1093/bja/aem308 . 17977860 10.1093/bja/aem308

[32] Bradley MM, Silakowski T, Lang PJ. Fear of pain and defensive activation. Pain. 2008;137(1):156–63. Epub 2007/10/02. doi: 10.1016/j.pain.2007.08.027 ; 17904289 10.1016/j.pain.2007.08.027 PMC2519040

[33] Moseley GL, Arntz A. The context of a noxious stimulus affects the pain it evokes. Pain. 2007;133(1–3):64–71. doi: 10.1016/j.pain.2007.03.002 17449180 10.1016/j.pain.2007.03.002

[34] Thibodeau MA, Welch PG, Katz J, Asmundson GJG. Pain-related anxiety influences pain perception differently in men and women: A quantitative sensory test across thermal pain modalities. Pain. 2013;154(3):419–26. doi: 10.1016/j.pain.2012.12.001 23337291 10.1016/j.pain.2012.12.001

[35] Gracely RH, Lota L, Walter DJ, Dubner R. A multiple random staircase method of psychophysical pain assessment. Pain. 1988;32(1):55–63. 3340422 10.1016/0304-3959(88)90023-1

[36] Strigo IA, Matthews SC, Simmons AN, Oberndorfer T, Klabunde M, Reinhardt LE, et al Altered insula activation during pain anticipation in individuals recovered from anorexia nervosa: evidence of interoceptive dysregulation. The International journal of eating disorders. 2013;46(1):23–33. Epub 2012/07/28. doi: 10.1002/eat.22045 ; 22836447 10.1002/eat.22045 PMC3507323

[37] Lin CS, Hsieh JC, Yeh TC, Lee SY, Niddam DM. Functional dissociation within insular cortex: the effect of pre-stimulus anxiety on pain. Brain research. 2013;1493:40–7. Epub 2012/12/04. doi: 10.1016/j.brainres.2012.11.035 . 23200718 10.1016/j.brainres.2012.11.035

[38] Price DD, McGrath PA, Rafii A, Buckingham B. The validation of visual analogue scales as ratio scale measures for chronic and experimental pain. Pain. 1983;17(1):45–56. Epub 1983/09/01. doi: 10.1016/0304-3959(83)90126-4 . 6226917 10.1016/0304-3959(83)90126-4

[39] Beck AT, Steer R, Brown G. Beck depression inventory. San Antonio, TX: The psychological corporation; 1996.

[40] Beck AT, Steer RA. Manual for the Beck anxiety inventory. San Antonio, TX: Psychological Corporation; 1990.

[41] Dworkin RH, Turk DC, Wyrwich KW, Beaton D, Cleeland CS, Farrar JT, et al Interpreting the Clinical Importance of Treatment Outcomes in Chronic Pain Clinical Trials: IMMPACT Recommendations. The Journal of Pain. 2008;9(2):105–21. doi: 10.1016/j.jpain.2007.09.005 18055266 10.1016/j.jpain.2007.09.005

[42] Bair MJ, Robinson RL, Katon W, Kroenke K. Depression and pain comorbidity: A literature review. Archives of Internal Medicine. 2003;163(20):2433–45. doi: 10.1001/archinte.163.20.2433 14609780 10.1001/archinte.163.20.2433

[43] Tracy LM, Georgiou-Karistianis N, Gibson SJ, Giummarra MJ. Location, location, location: Variation in sensitivity to pain across the body. European Journal of Pain. 2016;20(10):1721–9. doi: 10.1002/ejp.895 27221216 10.1002/ejp.895

[44] Dawson ME, Schell AM, Filion D. The electrodermal response system In: Cacioppo JT, Tassinary LG, editors. Principles of psychophysiology: Physical, social, and inferential elements. 3rd ed New York: Cambridge University Press; 2007 p. 159–81.

[45] Tabachnick BG, Fidell LS. Using multivariate statistics. 5th ed Boston: Pearson Education Inc; 2007.

[46] Field A. Discovering statistics using IBM SPSS Statistics and sex and drugs and rock 'n' roll. 4th ed London: Sage; 2013.

[47] Dunn OJ. Multiple comparisons among means. Journal of the American Statistical Association. 1961;56(293):52–64.

[48] Glass GV, Peckham PD, Sanders JR. Consequences of failure to meet assumptions underlying fixed effects analyses of variance and covariance. Review of Educational Research. 1972;42(3):237–88. doi: 10.3102/00346543042003237

[49] Harwell MR, Rubinstein EN, Hayes WS, Olds CC. Summarizing Monte Carlo results in methodological research: the one- and two-factor fixed effects ANOVA cases. Journal of Education and Behavioral Statistics. 1992;17(4):315–39. doi: 10.3102/10769986017004315

[50] Lix LM, Keselman JC, Keselman HJ. Consequences of assumption violations revisited: A quantitative review of alternatives to the one-way analysis of variance F test. Review of Educational Research. 1996;66(4):579–619.

[51] Girden ER. ANOVA: Repeated measures Sage University Papers Series on Quantitative Applications in the Social Sciences. 07–084. Newbury Park, CA: Sage; 1992.

[52] Lang EV, Hatsiopoulou O, Koch T, Berbaum K, Lutgendorf S, Kettenmann E, et al Can words hurt? Patient-provider interactions during invasive procedures. Pain. 2005;114(1–2):303–9. Epub 2005/03/01. doi: 10.1016/j.pain.2004.12.028 . 15733657 10.1016/j.pain.2004.12.028

[53] Dalton KM, Kalin NH, Grist TM, Davidson RJ. Neural-cardiac coupling in threat-evoked anxiety. Journal of cognitive neuroscience. 2005;17(6):969–80. Epub 2005/06/23. doi: 10.1162/0898929054021094 . 15969913 10.1162/0898929054021094

[54] Davis M, Falls WA, Campeau S, Kim M. Fear-potentiated startle: a neural and pharmacological analysis. Behavioural brain research. 1993;58(1–2):175–98. Epub 1993/12/20. . 8136044 10.1016/0166-4328(93)90102-v

[55] Phelps EA, O'Connor KJ, Gatenby JC, Gore JC, Grillon C, Davis M. Activation of the left amygdala to a cognitive representation of fear. Nature neuroscience. 2001;4(4):437–41. Epub 2001/03/29. doi: 10.1038/86110 . 11276236 10.1038/86110

[56] Rhudy JL, Meagher MW. Fear and anxiety: divergent effects on human pain thresholds. Pain. 2000;84(1):65–75. Epub 1999/12/22. doi: 10.1016/S0304-3959(99)00183-9 . 10601674 10.1016/S0304-3959(99)00183-9

[57] Barrett LF, Simmons WK. Interoceptive predictions in the brain. Nature reviews Neuroscience. 2015;16(7):419–29. Epub 2015/05/29. doi: 10.1038/nrn3950 ; 26016744 10.1038/nrn3950 PMC4731102

[58] Seth AK. Interoceptive inference, emotion, and the embodied self. Trends in cognitive sciences. 2013;17(11):565–73. Epub 2013/10/16. doi: 10.1016/j.tics.2013.09.007 . 24126130 10.1016/j.tics.2013.09.007

[59] Seth AK, Suzuki K, Critchley HD. An interoceptive predictive coding model of conscious presence. Frontiers in psychology. 2011;2:395 Epub 2012/02/01. doi: 10.3389/fpsyg.2011.00395 ; 22291673 10.3389/fpsyg.2011.00395 PMC3254200

[60] Rao RP, Ballard DH. Predictive coding in the visual cortex: a functional interpretation of some extra-classical receptive-field effects. Nature neuroscience. 1999;2(1):79–87. Epub 1999/04/09. doi: 10.1038/4580 . 10195184 10.1038/4580

[61] Hawkins J, Blakeslee S. On Intellegence. New York, NY: Owl Books Henry Holt and Company; 2004.

[62] Berna C, Leknes S, Holmes EA, Edwards RR, Goodwin GM, Tracey I. Induction of depressed mood disrupts emotion regulation neurocircuitry and enhances pain unpleasantness. Biological psychiatry. 2010;67(11):1083–90. Epub 2010/03/23. doi: 10.1016/j.biopsych.2010.01.014 . 20303069 10.1016/j.biopsych.2010.01.014

[63] Heller PH, Perry F, Naifeh K, Gordon NC, Wachter-Shikura N, Levine J. Cardiovascular autonomic response during preoperative stress and postoperative pain. Pain. 1984;18(1):33–40. Epub 1984/01/01. . 6709377 10.1016/0304-3959(84)90124-6

[64] Destrebecqz A, Peigneux P. Methods for studying unconscious learning. Progress in brain research. 2005;150:69–80. Epub 2005/09/28. doi: 10.1016/S0079-6123(05)50006-2 . 16186016 10.1016/S0079-6123(05)50006-2

