# Supplementary material for: Effects of explicit cueing and ambiguity on the anticipation and experience of a painful thermal stimulus
Source: PLoS One. 2017 Aug 23;12(8):e0183650. doi: 10.1371/journal.pone.0183650 (PMC5568281; doi:10.1371/journal.pone.0183650)
Supplement: S1 Table — (DOCX) [file pone.0183650.s005.docx]

**S1 Table. Instructions provided to participants**

| *Hint Condition:*  In this experiment you will be presented with a series of cues, with painful thermal stimuli following each cue. The colour of the cue will vary throughout the experiment, and will relate to the intensity of the stimulus that is delivered. Examples of the cues are presented below.   \| **Cue** \|  \| **Intensity** \| \| --- \| --- \| --- \| \| 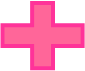 \|  \| None \| \| 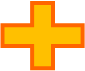 \|  \| Low \| \| 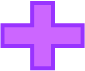 \|  \| High \| \| 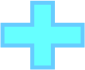 \|  \| Unknown \|   Following each stimulus you will be asked a series of questions relating to how you experienced and perceived the cue and its paired stimulus. |
| --- | --- | --- | --- | --- | --- | --- | --- | --- | --- | --- | --- | --- | --- | --- | --- |
|  |
| *No Hint Condition:*  In this experiment you will be presented with a series of cues, with thermal stimuli following each cue. Following each stimulus you will be asked a series of questions relating to how you experienced and perceived the cue and its paired stimulus. |

**Notes.** The “Hint” participants were informed that the colour of the cue would vary throughout the experiment, and that the colour would relate to the intensity of the stimulus that would be delivered, and were provided with a diagram of the visual cues and the paired stimulus intensity (see Supplementary Table A for a copy of the instructions provided to participants). Participants were unaware that there was an alternative set of instructions. Before the start of the experimental task, five training trials were performed to familiarise participants with the process and timing of a trial, as well as the rating procedure. The visual cues used in the training trials were different to those used in the real trials and thermal stimuli were not delivered during the training trials. Participants were simply told when the stimulus would be delivered. Training trials continued until the participant was comfortable and confident with the experimental procedure.
