# Supplementary material for: Effects of explicit cueing and ambiguity on the anticipation and experience of a painful thermal stimulus
Source: PLoS One. 2017 Aug 23;12(8):e0183650. doi: 10.1371/journal.pone.0183650 (PMC5568281; doi:10.1371/journal.pone.0183650)
Supplement: S2 Table — (DOCX) [file pone.0183650.s006.docx]

**S2 Table.** **Summary of main and interaction effects for subjective anxiety ratings**

|  | **Df** | **F** | **P** | **Effect Size** |
| --- | --- | --- | --- | --- |
| GROUP | 1, 49 | 5.32 | .03 | .10 |
| **CUE** | **1.82, 89.28** | **28.54** | **< .001** | **.37** |
| BLOCK | 1.32, 64.42 | 0.26 | .68 | .01 |
| **CUE x GROUP** | **1.82, 89.28** | **9.60** | **< .001** | **.16** |
| BLOCK x GROUP | 1.32, 64.42 | 0.26 | .67 | .01 |
| **CUE x BLOCK** | **2.86, 140.30** | **8.27** | **< .001** | **.14** |
| CUE x BLOCK x GROUP | 2.86, 140.30 | 1.59 | .20 | .03 |

**Note:** This table contains a summary of main and interaction effects from a mixed 2 x 3 x 2 x 3 repeated measures ANOVA, with GROUP (Hint/No Hint) as the between-subjects factor, and the BLOCK (1/2/3), the NATURE (Non-ambiguous/Ambiguous) and the TEMPERATURE of the stimulus (45 °C/41 °C/32 °C) as within-subjects factors. Significant interactions are highlighted in **bolded** text. df = degrees of freedom. Effect size reported as partial eta squared.
