# Supplementary material for: Effects of explicit cueing and ambiguity on the anticipation and experience of a painful thermal stimulus
Source: PLoS One. 2017 Aug 23;12(8):e0183650. doi: 10.1371/journal.pone.0183650 (PMC5568281; doi:10.1371/journal.pone.0183650)
Supplement: S3 Table — (DOCX) [file pone.0183650.s007.docx]

**S3 Table.** **Summary of main and interaction effects for anticipatory heart rate response**

|  | **Df** | **F** | **P** | **Effect Size** |
| --- | --- | --- | --- | --- |
| GROUP | 1, 47 | 2.06 | .16 | .04 |
| **CUE** | **3, 141** | **82.08** | **< .001** | **.64** |
| BLOCK | 2, 94 | 2.05 | .14 | .04 |
| CUE x GROUP | 3, 141 | 0.34 | .80 | .01 |
| BLOCK x GROUP | 2, 94 | 0.23 | .79 | .01 |
| CUE x BLOCK | 6, 282 | 0.41 | .87 | .01 |
| CUE x BLOCK x GROUP | 6, 282 | 1.30 | .26 | .03 |

**Note:** This table contains a summary of main and interaction effects from a mixed 2 x 3 x 2 x 3 repeated measures ANOVA, with GROUP (Hint/No Hint) as the between-subjects factor, and the BLOCK (1/2/3), the NATURE (Non-ambiguous/Ambiguous) and the TEMPERATURE of the stimulus (45 °C/41 °C/32 °C) as within-subjects factors. Significant interactions are highlighted in **bolded** text. df = degrees of freedom. Effect size reported as partial eta squared.
