# Supplementary material for: Effects of explicit cueing and ambiguity on the anticipation and experience of a painful thermal stimulus
Source: PLoS One. 2017 Aug 23;12(8):e0183650. doi: 10.1371/journal.pone.0183650 (PMC5568281; doi:10.1371/journal.pone.0183650)
Supplement: S5 Table — (DOCX) [file pone.0183650.s009.docx]

**S5 Table.** **Summary of main and interaction effects for pain intensity ratings**

|  | **df** | **F** | **P** | **Effect Size** |
| --- | --- | --- | --- | --- |
| GROUP | 1, 49 | 0.06 | .82 | < .01 |
| NATURE | 1, 49 | 1.32 | .26 | .03 |
| **TEMPERATURE** | **1.27, 62.02** | **275.81** | **< .001** | **.85** |
| **BLOCK** | **1.59, 77.97** | **7.84** | **.002** | **.14** |
| NATURE x GROUP | 1, 49 | 1.61 | .21 | .03 |
| TEMPERATURE x GROUP | 1.27, 62.02 | 0.22 | .70 | < .01 |
| BLOCK x GROUP | 1.59, 77.97 | 0.93 | .38 | .02 |
| **NATURE x TEMPERATURE** | **2, 98** | **11.04** | **< .001** | **.18** |
| **NATURE x TEMPERATURE x GROUP** | **2, 98** | **3.54** | **.03** | **.07** |
| **NATURE x BLOCK** | **2, 98** | **9.08** | **< .001** | **.16** |
| NATURE x BLOCK x GROUP | 2, 98 | 0.67 | .52 | .01 |
| **TEMPERATURE x BLOCK** | **3.46, 169.75** | **8.21** | **< .001** | **.14** |
| TEMPERATURE x BLOCK x GROUP | 3.46, 169.75 | 1.78 | .14 | .04 |
| **NATURE x TEMPERATURE x BLOCK** | **3.48, 170.74** | **13.73** | **< .001** | **.22** |
| NATURE x TEMPERATURE x BLOCK x GROUP | 3.48, 170.74 | 0.45 | .75 | .01 |

**Note:** This table contains a summary of main and interaction effects from a mixed 2 x 3 x 2 x 3 repeated measures ANOVA, with GROUP (Hint/No Hint) as the between-subjects factor, and the BLOCK (1/2/3), the NATURE (Non-ambiguous/Ambiguous) and the TEMPERATURE of the stimulus (45 °C/41 °C/32 °C) as within-subjects factors. Significant interactions are highlighted in **bolded** text. df = degrees of freedom. Effect size reported as partial eta squared.
