# Supplementary material for: Effects of explicit cueing and ambiguity on the anticipation and experience of a painful thermal stimulus
Source: PLoS One. 2017 Aug 23;12(8):e0183650. doi: 10.1371/journal.pone.0183650 (PMC5568281; doi:10.1371/journal.pone.0183650)
Supplement: S6 Table — (DOCX) [file pone.0183650.s010.docx]

**S6 Table.** Summary of Main and Interaction Effects for Pain Unpleasantness Ratings

|  | **df** | **F** | **P** | **Effect Size** |
| --- | --- | --- | --- | --- |
| GROUP | 1, 49 | 0.00 | .99 | .00 |
| NATURE | 1, 49 | 0.62 | .44 | .01 |
| **TEMPERATURE** | **1.22, 59.76** | **172.16** | **< .001** | **.78** |
| BLOCK | 1.62, 79.19 | 2.72 | .08 | .05 |
| NATURE x GROUP | 1, 49 | 3.28 | .08 | .06 |
| TEMPERATURE x GROUP | 1.22, 59.76 | 0.08 | .82 | < .01 |
| BLOCK x GROUP | 1.62, 79.19 | 1.37 | .26 | .03 |
| NATURE x TEMPERATURE | 1.64, 80.23 | 2.98 | .07 | .06 |
| NATURE x TEMPERATURE x GROUP | 1.64, 80.23 | 1.52 | .23 | .03 |
| **NATURE x BLOCK** | **1.72, 84.02** | **7.33** | **.002** | **.13** |
| NATURE x BLOCK x GROUP | 1.72, 84.02 | 0.34 | .68 | .001 |
| **TEMPERATURE x BLOCK** | **2.73, 133.98** | **8.47** | **< .001** | **.15** |
| TEMPERATURE x BLOCK x GROUP | 2.73, 133.98 | 1.59 | .20 | .03 |
| **NATURE x TEMPERATURE x BLOCK** | **3.36, 164.85** | **10.45** | **< .001** | **.18** |
| NATURE x TEMPERATURE x BLOCK x GROUP | 3.36, 164.85 | 0.45 | .74 | .01 |

**Note:** This table contains a summary of main and interaction effects from a mixed 2 x 3 x 2 x 3 repeated measures ANOVA, with GROUP (Hint/No Hint) as the between-subjects factor, and the BLOCK (1/2/3), the NATURE (Non-ambiguous/Ambiguous) and the TEMPERATURE of the stimulus (45 °C/41 °C/32 °C) as within-subjects factors. Significant interactions are highlighted in **bolded** text. df = degrees of freedom. Effect size reported as partial eta squared.
