# Supplementary material for: Effects of explicit cueing and ambiguity on the anticipation and experience of a painful thermal stimulus
Source: PLoS One. 2017 Aug 23;12(8):e0183650. doi: 10.1371/journal.pone.0183650 (PMC5568281; doi:10.1371/journal.pone.0183650)
Supplement: S7 Table — (DOCX) [file pone.0183650.s011.docx]

**S7 Table.** **Summary of main and interaction effects for stimulus heart rate response**

|  | **df** | **F** | **P** | **Effect Size** |
| --- | --- | --- | --- | --- |
| GROUP | 1, 47 | 0.54 | .47 | .01 |
| **NATURE** | **1, 47** | **4.16** | **.05** | **.08** |
| TEMPERATURE | 1.76, 82.78 | 2.21 | .13 | .04 |
| **BLOCK** | **2, 94** | **8.03** | **< .001** | **.15** |
| NATURE x GROUP | 1, 47 | 0.80 | .38 | .02 |
| TEMPERATURE x GROUP | 1.76, 82.78 | 1.27 | .28 | .03 |
| BLOCK x GROUP | 2, 94 | 1.42 | .25 | .03 |
| NATURE x TEMPERATURE | 2, 94 | 0.14 | .87 | < .01 |
| NATURE x TEMPERATURE x GROUP | 2, 94 | 0.77 | .46 | .02 |
| NATURE x BLOCK | 2, 94 | 2.08 | .13 | .04 |
| NATURE x BLOCK x GROUP | 2, 94 | 0.07 | .93 | < .01 |
| TEMPERATURE x BLOCK | 3.46, 162.58 | 1.64 | .18 | .03 |
| **TEMPERATURE x BLOCK x GROUP** | **3.46, 162.58** | **4.12** | **.005** | **.08** |
| NATURE x TEMPERATURE x BLOCK | 4, 188 | 0.88 | .48 | .02 |
| NATURE x TEMPERATURE x BLOCK x GROUP | 4, 188 | 0.88 | .48 | .02 |

**Note:** This table contains a summary of main and interaction effects from a mixed 2 x 3 x 2 x 3 repeated measures ANOVA, with GROUP (Hint/No Hint) as the between-subjects factor, and the BLOCK (1/2/3), the NATURE (Non-ambiguous/Ambiguous) and the TEMPERATURE of the stimulus (45 °C/41 °C/32 °C) as within-subjects factors. Significant interactions are highlighted in **bolded** text. df = degrees of freedom. Effect size reported as partial eta squared.
