# Supplementary material for: Effects of explicit cueing and ambiguity on the anticipation and experience of a painful thermal stimulus
Source: PLoS One. 2017 Aug 23;12(8):e0183650. doi: 10.1371/journal.pone.0183650 (PMC5568281; doi:10.1371/journal.pone.0183650)
Supplement: S8 Table — (DOCX) [file pone.0183650.s012.docx]

**S8 Table.** **Summary of main and interaction effects for stimulus skin conductance response**

|  | **df** | **F** | **P** | **Effect Size** |
| --- | --- | --- | --- | --- |
| GROUP | 1, 47 | 2.49 | .12 | .05 |
| NATURE | 1, 47 | 0.02 | .88 | < .01 |
| **TEMPERATURE** | **1.07, 50.43** | **41.52** | **< .001** | **.47** |
| **BLOCK** | **1.65, 77.64** | **6.56** | **.004** | **.12** |
| NATURE x GROUP | 1, 47 | 0.10 | .76 | < .01 |
| TEMPERATURE x GROUP | 1.07, 50.43 | 1.46 | .24 | .03 |
| BLOCK x GROUP | 1.65, 77.64 | 2.72 | .08 | .06 |
| **NATURE x TEMPERATURE** | **2, 94** | **4.09** | **.02** | **.08** |
| NATURE x TEMPERATURE x GROUP | 2, 94 | 2.66 | .08 | .05 |
| NATURE x BLOCK | 1.85, 86.75 | 6.76 | .002 | .13 |
| NATURE x BLOCK x GROUP | 1.85, 86.75 | 0.50 | .60 | .01 |
| TEMPERATURE x BLOCK | 2.17, 102.16 | 2.45 | .09 | .05 |
| **TEMPERATURE x BLOCK x GROUP** | **2.17, 102.16** | **3.97** | **.019** | **.08** |
| **NATURE x TEMPERATURE x BLOCK** | **3.47, 162.89** | **7.71** | **< .001** | **.14** |
| NATURE x TEMPERATURE x BLOCK x GROUP | 3.47, 162.89 | 0.67 | .59 | .01 |

**Note:** This table contains a summary of main and interaction effects from a mixed 2 x 3 x 2 x 3 repeated measures ANOVA, with GROUP (Hint/No Hint) as the between-subjects factor, and the BLOCK (1/2/3), the NATURE (Non-ambiguous/Ambiguous) and the TEMPERATURE of the stimulus (45 °C/41 °C/32 °C) as within-subjects factors. Significant interactions are highlighted in **bolded** text. df = degrees of freedom. Effect size reported as partial eta squared.
