# Supplementary material for: Effects of explicit cueing and ambiguity on the anticipation and experience of a painful thermal stimulus
Source: PLoS One. 2017 Aug 23;12(8):e0183650. doi: 10.1371/journal.pone.0183650 (PMC5568281; doi:10.1371/journal.pone.0183650)
Supplement: S1 File — Summary of non-parametric statistical analyses. (DOCX) [file pone.0183650.s013.docx]

**S1 File. Evidence for Consistent Effects across Non-Parametric Comparisons.**

Subjective Anxiety Ratings – Main effect of GROUP

A Mann Whitney *U* test indicated that the “Hint” participants reported higher levels of subjective anxiety (*Mean Rank* = 31.23, *n* = 26) compared to the “No Hint” participants (*Mean Rank* = 21.77, *n* = 26) following the presentation of the visual cue, *U* = 215, *z* = -2.25, *p* = .024, Cohen’s *r* = .31.

Subjective Anxiety Ratings – CUE x GROUP Interaction

A series of Wilcoxon signed rank tests indicated that for the “Hint” participants, the Purple cue (always preceding a 45 °C thermal heat stimulus; Sum of Ranks = 284, 270.50, and 274, respectively) yielded higher ratings of anxiety than the Orange (always preceding a 41 °C thermal heat stimulus; Sum of Ranks = 16, *p* < .001, *r* = .75), Pink (always preceding a 32 °C thermal heat stimulus; Sum of Ranks = 5.50, *p* < .001, *r* = .79), and Blue (always preceding a thermal stimulus of ambiguous temperature; Sum of Ranks = 51, *p* = .003, *r* = .59) cues. The Blue cue (Sum of Ranks = 192 and 251, respectively) yielded higher ratings of anxiety than the Orange (Sum of Ranks = 39, *p* = .008, *r* = .52) and Pink (Sum of Ranks = 25, *p* = .001, *r* = .67) cues. The Orange cue (Sum of Ranks = 188.50) yielded higher ratings of anxiety than the Pink cue (Sum of Ranks = 21.50, *p* = .002, *r* = .61).
