# Supplementary material for: Effects of explicit cueing and ambiguity on the anticipation and experience of a painful thermal stimulus
Source: PLoS One. 2017 Aug 23;12(8):e0183650. doi: 10.1371/journal.pone.0183650 (PMC5568281; doi:10.1371/journal.pone.0183650)
Supplement: S2 File — Summary of statistical analyses using when trimmed means. (DOCX) [file pone.0183650.s014.docx]

**S2 File. Evidence for Consistent Effects when using Trimmed Means.**

Trimmed means (i.e., removing the lowest and highest value from each data set) were calculated for each of the three non-ambiguous cues (i.e., high pain, low pain, and innocuous sensation) and the ambiguous cue (unknown pain). The effect of explicit cuing on anxiety was determined with a mixed 2 x 3 x 4 repeated measures analysis of variance (ANOVA), with GROUP (Hint/No Hint) as the between-subjects factor, and with the BLOCK of the pain anxiety task (1/2/3) and the colour of the CUE (Purple/Orange/Pink/Blue) as within-subjects factors.

Subjective Anxiety Ratings

There was a main effect of GROUP on subjective anxiety ratings; *F*(1, 49) = 5.15, *p* = .028, partial eta squared (*η^2^_p_*) = .10. The “Hint” participants reported higher levels of subjective anxiety (*M* = 2.60, SE = 0.37) compared to the “No Hint” participants (*M* = 1.42, SE = 0.37). There was also a main effect of CUE on subjective anxiety ratings; *F*(1.85, 90.81) = 27.77, *p* < .001, *η^2^_p_* = .36. The Purple cue (45 °C; *M* = 3.21, SE = 0.41) yielded higher ratings of subjective anxiety than the Orange (41 °C; *M* = 1.49, SE = 0.23, *p* < .001), Pink (32 °C; *M* = 1.09, SE = 0.24, *p* < .001) and Blue (ambiguous; *M* = 2.26, SE = 0.31, *p* = .001) cues. The Blue cue yielded higher ratings of subjective anxiety than the Orange (*p* = .007) and Pink (*p* = .001) cues.
